# 3D Nanoflowers of Binary Metal‐Selenide for Improved Electrochemical Sensing and High‐Energy‐Density Energy Storage

**DOI:** 10.1002/smll.202505860

**Published:** 2025-07-11

**Authors:** Keerthika Devi Ramadhass, Chun Che Lin

**Affiliations:** ^1^ Institute of Organic and Polymeric Materials Research and Development Center for Smart Textile Technology National Taipei University of Technology Taipei 106 Taiwan (R.O.C)

**Keywords:** electrochemical sensors, nanoflowers, nilutamide, NiVSe, supercapacitors

## Abstract

Electrochemical sensing and energy storage devices are often hindered by the limited conductivity, low redox activity, and poor cycling stability of traditional electrode materials. To overcome these limitations, we report the design and synthesis of a novel 3D nanoflower‐like nickel vanadium selenide (NF‐NiVSe) architecture, formed by integrating nickel selenide (NiSe) nanoflakes and vanadium selenide (VSe) nanobelts. The bimetallic integration of Ni and V creates a hierarchically structured material with enhanced surface area, abundant electroactive sites, and efficient electron transport pathways. As an electrochemical sensor, NF‐NiVSe demonstrates outstanding performance in detecting the anticancer drug nilutamide (NLT), with a low reduction potential (−0.53 V), high current response (−56.31 µA), and an ultralow detection limit of 0.2 nM, achieving ≈99% recovery in real samples. In supercapacitor applications, NF‐NiVSe exhibits a high specific capacitance of 1695 F g⁻¹ at 1.5 A g⁻¹, excellent cycling retention (90% after 10,000 cycles), and 98% Coulombic efficiency. A symmetric NF‐NiVSe//NF‐NiVSe device achieves an energy density of 85 Wh kg⁻¹ at 1800 W kg⁻¹ and powers an LED, showcasing practical viability. The exceptional dual‐functionality is attributed to the synergistic redox activity of Ni and V and the unique nanoflower morphology, positioning NF‐NiVSe as a promising material for multifunctional applications.

## Graphical abstract

This study reports the hydrothermal synthesis of 3D NiVSe nanoflowers from NiSe nanoflakes and VSe nanobelts at 180 °C for 12 h. The hybrid structure enables dual functionality: ultrasensitive electrochemical detection of nilutamide with a detection limit of 0.2 nm, and high‐performance supercapacitor behavior with an energy density of 85 Wh kg⁻¹ at a power density of 1800 W kg⁻¹.

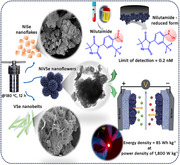

## 1 Introduction

The rapid advancement of electrochemical devices critically depends on the development of advanced active materials. Among emerging candidates, binary metal selenides (BMS) have gained increasing attention due to their high electrical conductivity, chemical stability, and redox activity,^[^ ^1^ ^]^ outperforming sulfur (S) and oxygen (O)‐based analogs owing to selenium's (Se) superior intrinsic conductivity (10^−3^ S m^−1^).^[^ ^2^ ^]^ By leveraging the synergistic effects of dual metal centers with Se, BMS significantly enhances electron transfer and catalytic efficiency,^[^ ^3^ ^]^ offering an effective strategy to overcome the limitations of conventional materials in both electrochemical sensing and supercapacitors. Traditional supercapacitor materials, including carbon‐based systems, single‐metal oxides, and layered double hydroxides (LDHs), suffer from drawbacks such as low specific capacitance, sluggish ion transport, and poor cycling stability. Layered double hydroxides (LDHs), particularly those based on Co and Ni, have garnered considerable interest as electrode materials for supercapacitors due to high theoretical capacitance, and rich redox chemistry.^[^ ^4^, ^5^, ^6^ ^]^ However, their practical deployment is often hindered by intrinsic limitations such as poor electrical conductivity, sluggish ion diffusion, and structural instability under extended cycling.^[^ ^7^, ^8^, ^9^ ^]^ For example, Co₁Ni₂(OH)₂‐based LDHs, though capable of achieving energy densities as high as 62.8 Wh kg⁻¹ and excellent retention (≈99%) over 25 000 cycles, still suffer from relatively moderate rate capabilities and require complex synthetic strategies or conductive additives to maintain performance stability.^[^ ^10^ ^]^ Similarly, hybrid systems such as Co─Ni LDH supported on macroporous carbon exhibit long‐term cycling stability (91.1% over 30 000 cycles), but at the cost of limited energy density (23.3 Wh kg⁻¹ at 15 kW kg⁻¹), suggesting a trade‐off between power delivery and energy storage.^[^ ^11^ ^]^

BMS effectively addresses these limitations by integrating hierarchical architectures, Se‐mediated conductivity, and synergistic redox‐active centers, resulting in superior specific capacitance, enhanced ion diffusion, and long‐term electrochemical stability. Electrochemical sensing of pharmaceutical residues also demands advanced materials with high sensitivity, selectivity, and operational stability. Nilutamide (NLT), an anti‐androgen drug used for treating advanced prostate cancer—the second most common cancer in men^[^ ^12^ ^]^ requires precise monitoring in biological fluids due to its severe side effects, including bone density loss, liver toxicity, and depression.^[^ ^13^ ^]^ Moreover, the environmental accumulation of NLT raises serious ecological concerns.^[^ ^14^ ^]^ Although methods such as micellar electrokinetic chromatography, spectrophotometry, and electrochemical techniques have been explored,^[^ ^15^, ^16^, ^17^ ^]^ electrochemical approaches remain the most promising due to their rapid response, selectivity, and cost‐effectiveness.^[^ ^14^, ^18^, ^19^ ^]^ However, existing sensors are often limited by poor sensitivity and stability, highlighting the urgent need for novel electrode materials with superior performance. Similarly, in the field of energy storage, the development of battery‐type electrode materials has gained attention for their high theoretical specific capacities, achieved through rapid and reversible redox reactions.^[^ ^20^ ^]^ Designing versatile electrode materials capable of addressing the challenges in both drug detection and energy storage represents a critical step toward advancing multifunctional electrochemical technologies.

Transition metal‐based selenides, including NiSe_2_, CoSe_2_, VSe_2_, and MoSe_2_, have demonstrated excellent performance across a range of electrochemical technologies, including supercapacitors,^[^ ^21^ ^]^ batteries,^[^ ^22^ ^]^ fuel cells,^[^ ^23^ ^]^ solar cells^[^ ^24^ ^]^ and electrochemical sensors^[^ ^14^, ^25^, ^26^, ^27^ ^]^ Their narrow band gaps and robust chemical stability enable high electrochemical activity and durability. Specifically, NiSe_2_ has been used for glucose sensing,^[^ ^28^ ^]^ and efficient electrocatalyst for enhanced H_2_ generation.^[^ ^29^ ^]^ While VSe_2_ nanoflakes have been employed for nitrobenzene detection,^[^ ^30^ ^]^ and efficient H_2_ evolution through defect engineering.^[^ ^31^ ^]^ The metallic behavior of VSe_2_, driven by V^4+^–V^4+^ coupling,^[^ ^32^ ^]^ enhances conductivity and facilitates rapid charge transfer, although its electrochemical stability is limited by the oxidation of V^4+^, to V^3+^ or V^5+^.^[^ ^33^ ^]^ Recent advances show that hybrid and bimetallic architectures improve the structural robustness and electrochemical performance by exploiting synergistic effects between dual‐metal centers.^[^ ^21^, ^34^, ^35^ ^]^ Despite these advances, Ni and V‐based bimetallic selenide nanostructures remain underexplored. Recent studies, such as battery‐type NiVSe developed by Xuan et al.^[^ ^20^ ^]^ and NiVSe/MXene nanocomposite by Cochran et al.^[^ ^36^ ^]^ have demonstrated promising applications in energy storage. However, to date, NiVSe has not been investigated for electrochemical sensing applications. Given its unique structural and electronic properties, such as d–p orbital hybridization, multiple accessible oxidation states, and high intrinsic conductivity,^[^ ^37^ ^]^ NiVSe offers an exciting opportunity for a dual‐functional device. Herein, we report the synthesis and characterization of NiSe nanoflakes, VSe nanobelts, and 3D NF‐NiVSe nanostructures. The NF‐NiVSe features a high surface‐to‐volume ratio and interconnected conductive networks, significantly outperforming monometallic counterparts in electrochemical performance. We explored the electrocatalytic behavior of NF‐NiVSe‐modified glassy carbon electrodes (NF‐NiVSe/GCE) for sensitive NLT detection and validated its performance in real biological samples. Furthermore, NF‐NiVSe exhibited a substantially higher specific capacitance than VSe and NiSe, demonstrating its exceptional potential as a dual‐functional material for both therapeutic monitoring and next‐generation energy storage devices.

## 2 Experimental Section

**Scheme 1:**
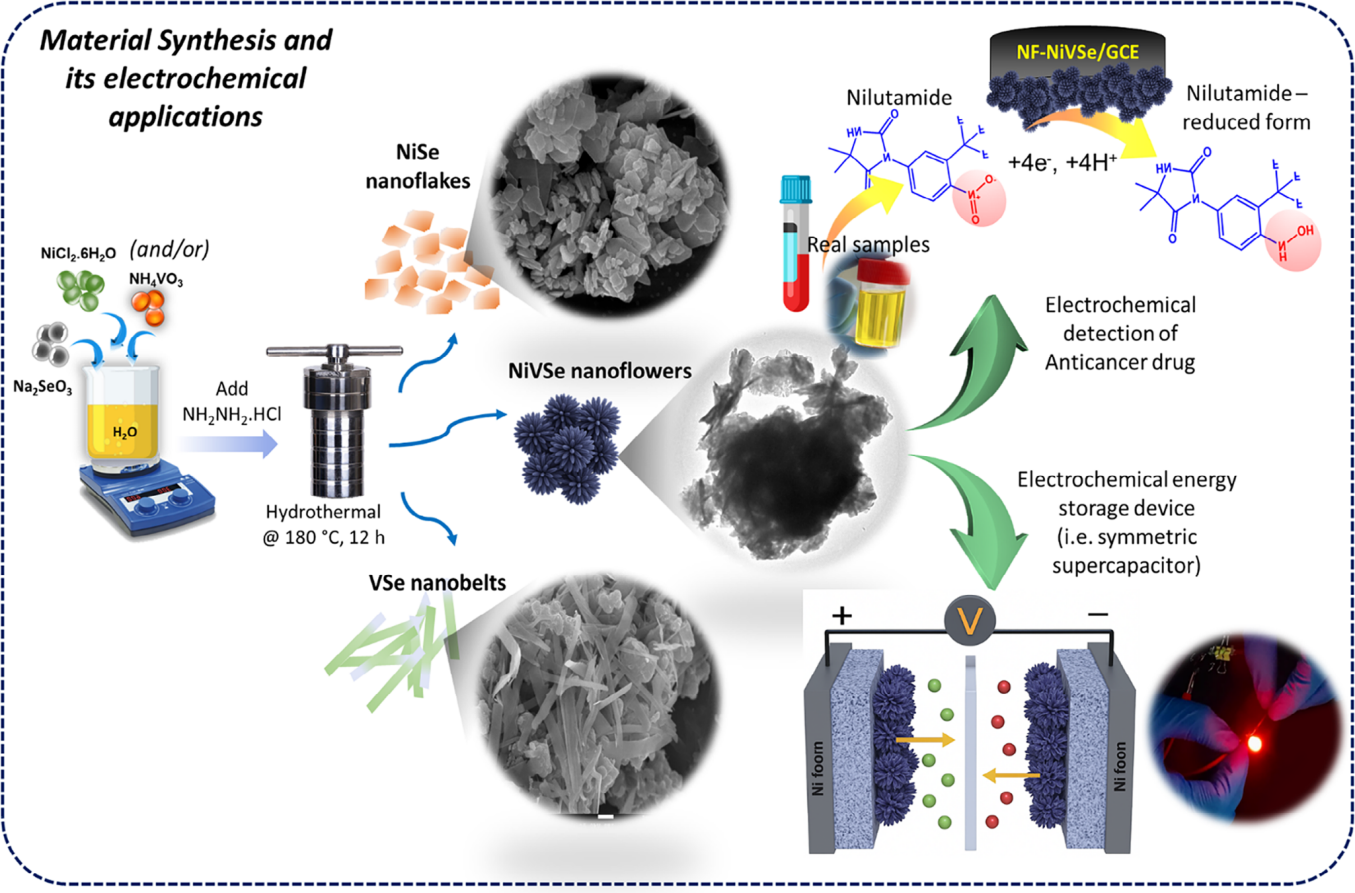
Schematic illustration of the synthesis, characterization, and multifunctional application of NF‐NiVSe nanostructures. A one‐pot hydrothermal approach was employed using precursor metal salts and selenium powder to synthesize flower‐like NiVSe nanostructures on nickel foam substrates. SEM and TEM analyses confirmed the successful formation of hierarchical nanoflowers. The synthesized NF‐NiVSe material was further explored for two applications: i) as an electrocatalytic sensing interface on GCE (glassy carbon electrode) for the detection of a target analyte, facilitated by redox‐active interactions, and ii) as symmetric electrodes in a supercapacitor device, demonstrating excellent electrochemical performance. The assembled device successfully powered a red LED, validating its energy storage capability and real‐world applicability.

Details on chemicals and reagents, synthesis, characterization, electrochemical measurements, and electrode fabrications are presented in the Supporting Information. The material synthesis procedure is graphically illustrated in **Scheme** **1**.

## 3 Results and Discussion

### 3.1 Structural and Morphological Analysis

**Figure 1:**
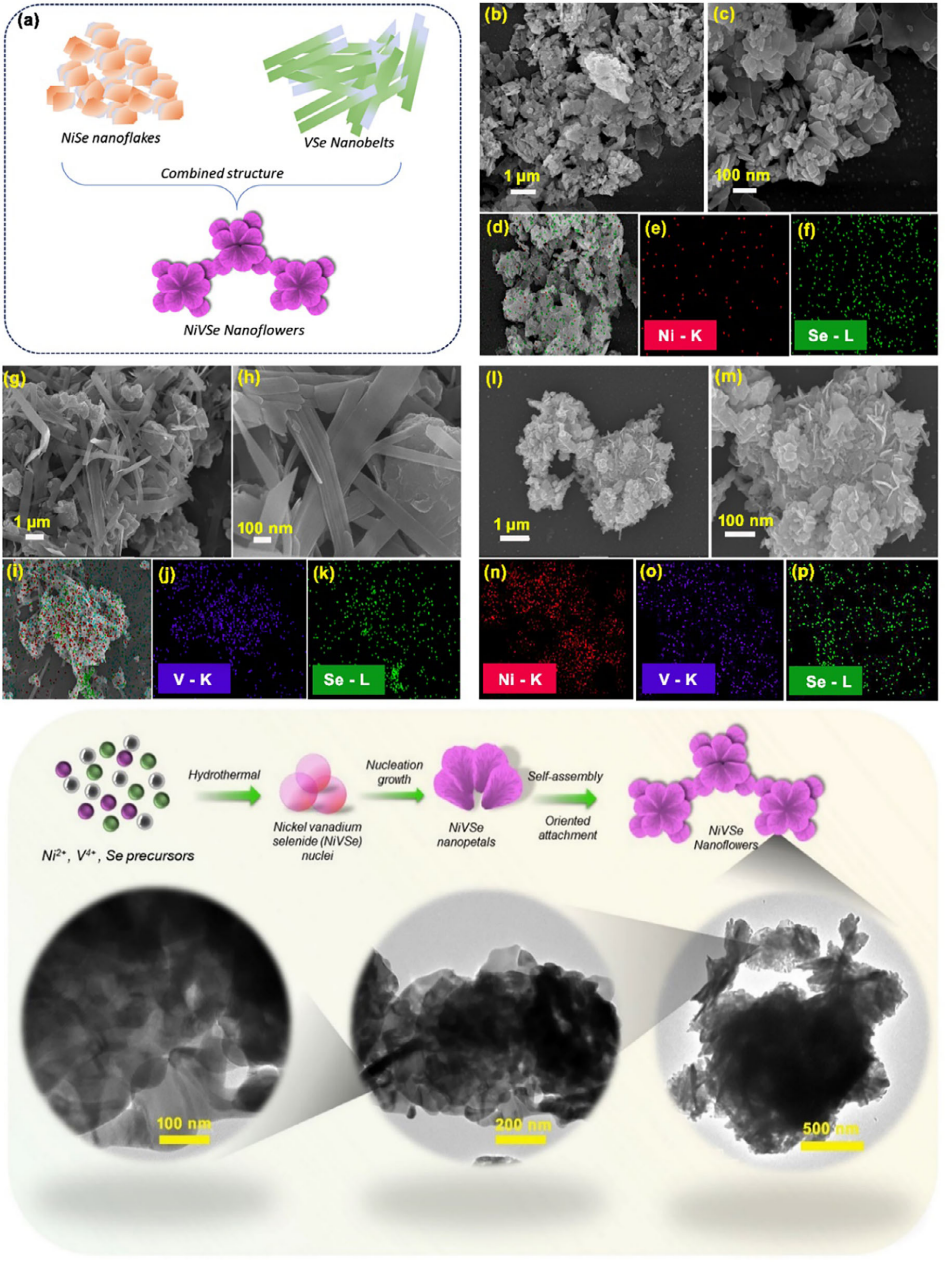
a) Schematic representation for the nanostructures of NiSe, VSe, and NF‐NiVSe. FE‐SEM images of NiSe nanoflakes at magnifications of b) 1 µm, and c) 100 nm. Elemental mapping of NiSe showing d) mixed elements, e) Ni, and f) Se. FE‐SEM images of VSe nanobelts at the magnifications of g) 1 µm, and h) 100 nm. Elemental mapping of VSe showing i) mixed elements, j) V, and k) Se. FE‐SEM images of NiVSe nanoflowers at the magnifications of l) 1 µm, and m) 100 nm. Elemental mapping results of NiVSe nanoflowers for the n) Ni, o) V, and p) Se. q) Schematic illustration of the proposed structural growth mechanism of NF‐NiVSe along with HR‐TEM images at varying magnifications to highlight the detailed structural features.

The surface morphology of the synthesized samples was investigated using various spectroscopic techniques. **Figure** **1**a schematically illustrates the morphologies: NiSe exhibits a nanoflake‐like structure, VSe forms a nanobelt‐like morphology, and NiVSe combines these features to create a hierarchical 3D nanoflower‐like morphology. FE‐SEM images of NiSe nanoflakes at different magnifications reveal densely packed nanosheets at low magnification (Figure 1b). At higher magnifications (Figure 1c), the sheers are observed as thin, stacked layers with sharp edges, measuring 70–80 nm in diameter. Elemental mapping (Figure 1d–f) shows a uniform distribution of nickel (Ni) and selenium (Se) across the nanoflakes, confirming their homogeneous composition. VSe nanobelts, displayed in Figure 1g,h, appear as long, continuous, ribbon‐like structures. The densely packed, intertwined belts provide a high surface area, advantageous for applications such as catalysis and energy storage. At higher magnification (100 nm scale), the smooth, well‐defined surfaces of the nanobelts suggest high crystalline quality. Elemental mapping (Figure 1i–k) confirms the even distribution of vanadium (V) and Se throughout the nanobelts, ensuring consistent catalytic performance and structural integrity.

The morphology of NiVSe nanoflowers (NF‐NiVSe) is illustrated in Figure 1l,m. While NiSe, and VSe exhibit 2D morphologies individually, the combination of Ni, V, and Se precursors during synthesis results in the formation of hierarchical 3D nanoflowers. Elemental mapping (Figure 1n–p) confirms the uniform distribution of Ni, V, and Se elements across the nanoflower structure, highlighting its compositional consistency and potential for enhanced electrochemical applications. To gain deeper insights into the structural features of NF‐NiVSe, HR‐TEM analysis was performed, as shown in Figure 1q, alongside the proposed growth mechanism. During synthesis, the precursors nucleated to form NiVSe, which subsequently developed into nanopetal‐like structures. These nanopetals likely self‐assembled and interconnected, leading to the formation of the nanoflower‐like morphology. These nanopetals likely self‐assembled and attached to one another, resulting in the formation of the nanoflower‐like morphology. The hierarchical structure of the nanoflowers becomes more evident at this stage, revealing the integration of the two precursor materials—NiSe nanoflakes and VSe nanobelts—into a unified nanostructure. The NiSe nuclei formed the core of the nanoflower, with their thin, plate‐like morphology contributing to the overall “petal‐like” appearance. This structural arrangement highlights the synergy between the components, resulting in a well‐organized and unique architecture.

**Figure 2:**
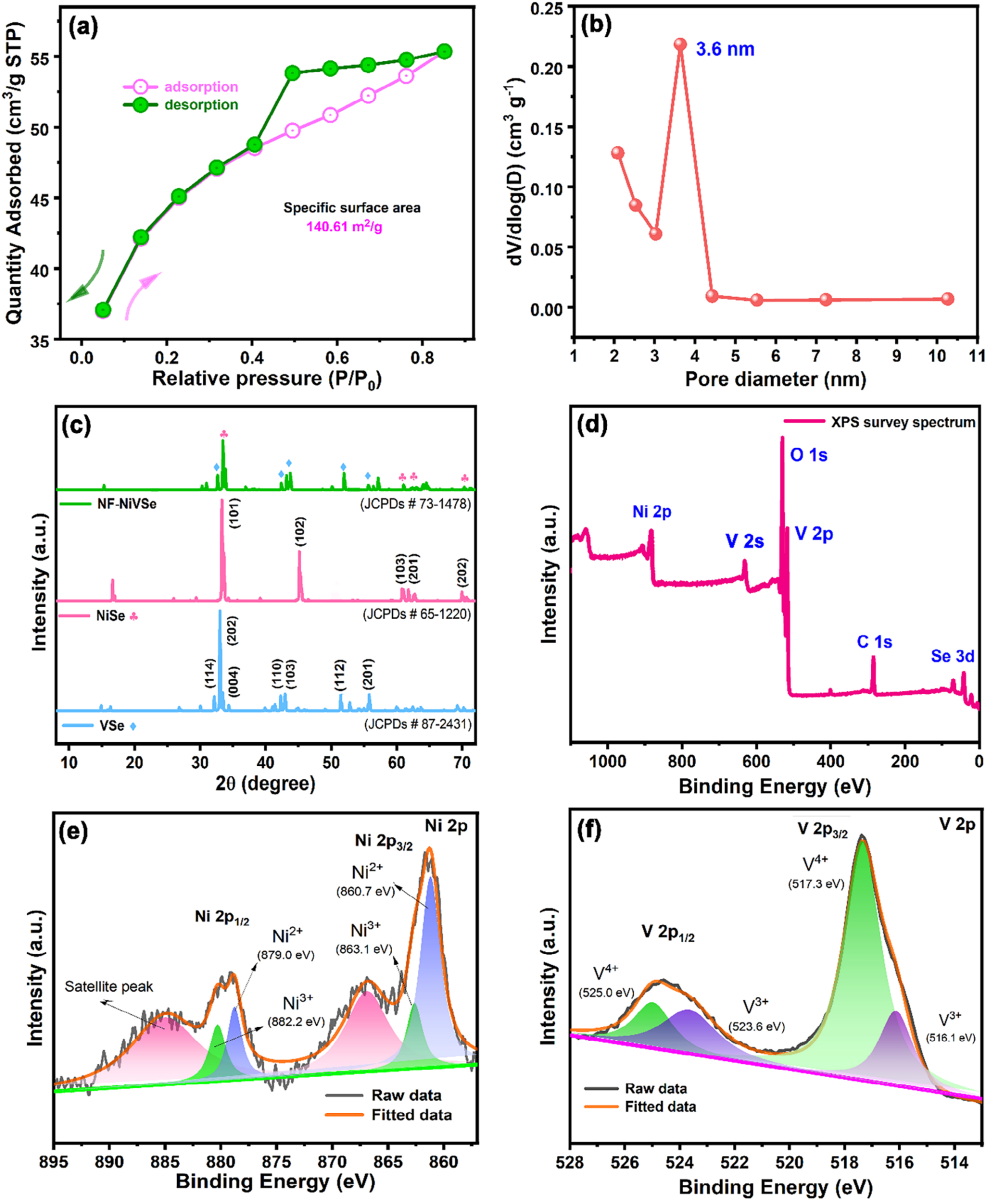
a) N_2_ adsorption/desorption isotherm plot for NF‐NiVSe, showing surface area and porosity, and b) corresponding pore‐size distribution. c) PXRD patterns of as‐synthesized NiSe, VSe, and NF‐NiVSe, displaying crystalline structures. d) XPS survey spectrum of NF‐NiVSe indicating elemental composition. High‐resolution XPS spectra of e) Ni 2p, and (f) V 2p, revealing oxidation states and chemical environments of Ni and V.

Meanwhile, the VSe nuclei interweave with the nanoflakes, adding an elongated, belt‐like dimension to the structure. This combination of morphologies creates a highly ordered, multilayered architecture, where the components are integrating seamlessly. The hierarchical assembly of thin layers and elongated belts enhances surface roughness and increases the number of available active sites, making the structure particularly advantageous for applications such as catalysis and electrochemical reactions, where high surface activity is desirable. The surface area pore size distribution of NF‐NiVSe were analyzed using N_2_ adsorption—desorption isotherms and the Barrett–Joyner–Halenda method. The isotherms (**Figure** **2**a) reveal a significant increase in N_2_ adsorption volume within the *P*/*P*_0_ range of 0.4–0.8, indicating a mesoporous structure. This increase, attributed to capillary condensation within the mesopores, is highlighted by the inflection point in this range, signaling pore filling. The average pore diameter was determined to be 3.6 nm (Figure 2b), with a specific surface area of 140.61 m^2 ^g^−1^. This high surface area and mesoporosity enhance the adsorption of analyte molecules and provide more active sites, which are essential for improving electrocatalytic activity. The hierarchical nanoflower structure also promotes efficient mass transport,^[^ ^38^, ^39^ ^]^ making NF‐NiVSe highly suitable for electrocatalysis.

Additionally, the combination of a large surface area and well‐defined pore structure positions NF‐NiVSe as a promising material for energy storage technologies, where high surface activity is essential for efficient performance. Figure 2c illustrates the PXRD analysis results, shedding light on the crystallinity and phase purity of VSe nanobelts, NiSe nanoflakes, and NF‐NiVSe nanostructures. The PXRD patterns reveal well‐defined crystalline phases for each sample, allowing the identification of characteristic diffraction peaks and their corresponding crystallographic planes. For the VSe nanobelts, significant diffraction peaks appear at 2*θ* values of 31.9°, 32.8°, 34.3°, 42.4°, 42.9°, 51.4°, and 55.5°, corresponding to the (114), (202), (004), (110), (103), (112), and (201) planes, respectively. These peaks align with the standard data for the hexagonal phase of V_3_Se_4_ (JCPDs# 87–2431),^[^ ^40^ ^]^ confirming a high degree of crystallinity with minimal structural defects and impurities. The sharp and intense peaks further confirm the phase purity of the VSe nanobelts.

Additionally, the presence of these crystallographic planes highlights the anisotropic growth of the VSe nanobelts, characterized by their long, ribbon‐like morphology, as observed in FE‐SEM results. The NiSe nanoflakes exhibit distinct diffraction peaks at 2*θ* values of 33.3°, 45°, 60.8°, 61.9°, and 70°, corresponding to the (101), (102), (103), (201), and (202) planes, respectively. These sharp and well‐defined peaks indicate a highly crystalline structure, consistent with the hexagonal phase of Ni_3_Se_4_ (JCPDs#73‐1478).^[^ ^41^ ^]^ The dominant reflections at low angles (101) and (102) suggest ordered growth, while the higher angle reflections (103), (201), and (202) represent well‐developed, high‐energy crystal facets. This confirms that the NiSe nanoflakes possess a well‐ordered hexagonal lattice and excellent phase purity. The NF‐NiVSe nanostructures exhibit diffraction peaks that combine distinct reflections of both NiSe and VSe phases, confirming the successful integration of these components into the composite nanoflower structure and in good alignment with the standard data of NiV_2_Se_4_ (JCPDS# 73–1478). Key reflections for NiSe include those at 2*θ* = 33.2° (101), 45.1° (102), and 70.0° (202), corresponding to the hexagonal phase of NiSe. Similarly, the characteristic peaks for VSe, appearing at 2*θ* = 28.6° (114), 30.1° (004), and 55.6° (201), represent the hexagonal phase of VSe. The presence of both sets of peaks in the NF‐NiVSe pattern suggests that the NiSe and VSe components retain their individual crystallographic phases within the combined nanoflower structure. Thereby, it is expected that it could show superior performance than their individual counterparts.

XPS analysis was conducted to investigate the chemical states of the NF‐NiVSe. A wide‐range energy survey scan (Figure 2d) confirmed the presence of Ni, V, Se, C, and O, with distinct signals observed at the binding energies (BEs) of 875, 520, 60, 284, and 535 eV, corresponding to Ni 2p, V 2p, Se 3d, C 1s and O 1s, respectively. The C 1s signal likely arises from instrument‐related carbon contamination during XPS analysis, while the O 1s signal is attributed to surface moisture adsorption. The high‐resolution Ni 2p spectrum (Figure 2e) was deconvoluted to reveal two prominent peaks at 860.7 and 879 eV, corresponding to Ni^2+^ ions. Additionally, two smaller peaks at 863.1 and 882.2 eV were identified, indicating the presence of Ni^3+^ ions,^[^ ^42^, ^43^ ^]^ which suggest a mixed oxidation state of Ni within the nanocomposite. Satellite peaks at 866.9 eV (Ni 2p_3/2_) and 885.7 eV (Ni 2p_1/2_) further support the presence of Ni in multiple oxidation states, reflecting the unique electronic environment of Ni in the NF‐NiVSe structure. Similarly, the high‐resolution V 2p spectrum (Figure 2f) revealed characteristic peaks for vanadium. The V 2p_3/2_ peak at 517.3 eV and the V 2p_1/2_ peak at 525 eV correspond to V^4+^. Another set of peaks at 516.1 eV (V 2p_3/2_) and 523.6 eV (V 2p_1/2_) represents V^3+^, indicating the coexistence of V^3+^ and V^4+^ oxidation states. This spin‐orbital splitting between the V 2p_1/2_ and V 2p_3/2_ peaks results in doublet peaks, confirming the mixed valence state of vanadium within the NF‐NiVSe nanostructure. These results highlight the complex electronic environment of the NF‐NiVSe nanocomposite, where both nickel and vanadium exist in multiple oxidation states. This dynamic surface chemistry likely enhances the electronic and catalytic properties of the material. The coexistence of Ni^2+^/Ni^3+^ and V^3+^/V^4+^ suggests that NF‐NiVSe possesses significant potential for electrocatalysis, where mixed‐valence states are known to play a critical role in enhancing performance.

### 3.2 Electrochemical Characterization

**Figure 3:**
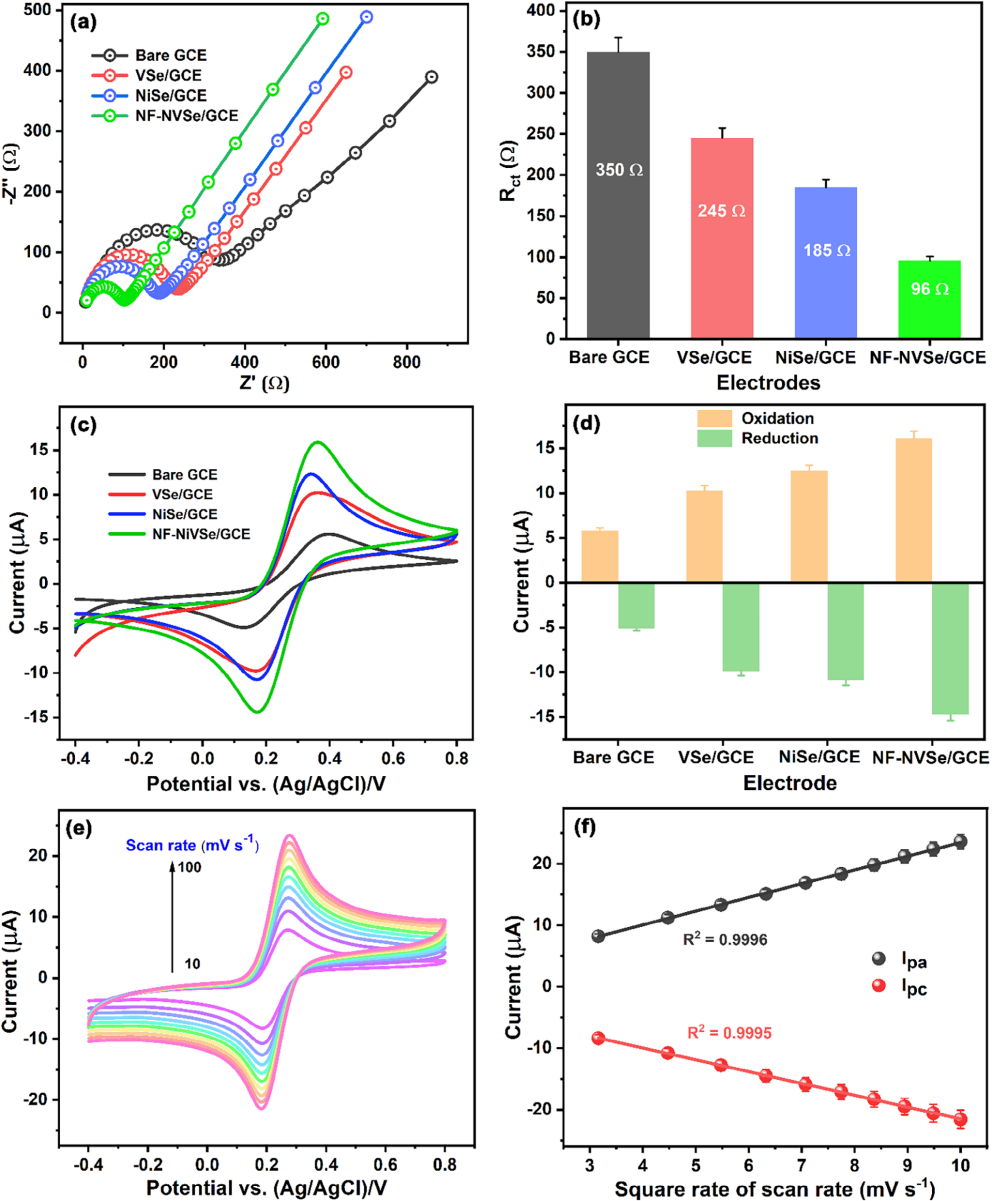
a) EIS spectra of bare and modified electrodes over a frequency range of 1 Hz to 100 kHz, illustrating changes in *R*_ct_ b) Bar chart comparing *R*_ct_ values for bare and modified electrodes. c) CVs of bare and modified electrodes at a scan rate of 50 mV s^−1^, showing a redox behavior, and d) corresponding bar chart comparing anodic and cathodic peak currents for bare and modified electrodes. e) CVs of NF‐NiVSe/GCE at varying scan rates (10–100 mV s^−1^) to evaluate the scan rate dependency and f) a linear plot of the square root of scan rate versus current, confirming diffusion‐controlled processes. All experiments were conducted in 0.1 m KCl containing 5 mm [Fe(CN)_6_]^3‐/4‐^ as the electrolyte.

The charge transfer resistance (*R_ct_*) at the electrode/electrolyte interface of the bare GCE and modified electrodes, including NiSe/GCE, VSe/GCE, and NF‐NiVSe/GCE, was analyzed using electrochemical impedance spectroscopy (EIS) in a three‐electrode configuration. A 0.1 m KCl solution containing 5 mm [Fe(CN)_6_]^3‐/4‐^ served as a supporting electrolyte (standard probe solution). The EIS profiles, presented as Nyquist plots (**Figure** **3**a), consistently displayed a semicircle feature at high frequencies followed by a linear region at low frequencies. The semicircles correspond to electron transfer events, with their diameters indicating the charge transfer resistance, while the linear region represents the Warburg diffusion of charge carriers.

The Nyquist plots were fitted using Randel's equivalent circuit model (Insert Figure 3a), which comprises the charge‐transfer resistance (*R_ct_*), series resistance (*R_s_*), constant phase element (*CPE*), and Warburg impedance (*Z_W_*). The *R_ct_* encompasses both the contact and intrinsic resistance of the material, and its value can be determined from the diameter of the semicircle. The *R_ct_* values for the bare GCE, NiSe/GCE, VSe/GCE, and NF‐NiVSe/GCE are 350, 245, 185, and 98 Ω, respectively (Figure 3b), with NF‐NiVSe/GCE exhibiting the lowest *R_ct_*, indicating superior electron transfer kinetics than the compared to the other modified electrodes. The 3D flower‐like structure of NF‐NiVSe offers abundant active sites and a larger surface area, enhancing electrolyte diffusion and electron transport. Further, the modified electrodes' charge transfer rate constant (ks) was calculated using Equation (1).

(1) $$R_{ct}=\frac{RT}{n^{2}F^{2}k_{s}C}$$

where, *R* (gas constant = 8.314 J mol^−1 ^K^−1^)*, T* (temperature = 25 °C)*, n* (number of electron transfer), *F* (Faraday constant = 96 485 C mol^−1^)*, C* (concentration), and *k_s_* (charge transfer rate). According to Equation (1), the *k*_s_ values for bare GCE, NiSe/GCE, VSe/GCE, and NF‐NiVSe/GCE were 3.8 × 10^−8^, 5.4 × 10^−8^, 7.2 × 10^−8^, and 1.9 × 10^−7^ cm s^−1^, respectively. These results confirm that NF‐NiVSe/GCE exhibits superior electron transfer reversibility and catalytic activity over other modified electrodes.

Cyclic voltammetry (CV) was conducted in a standard probe solution at a scan rate of 50 mV s^−1^ to evaluate both bare and modified electrodes' peak‐to‐peak potential difference (∆*Ep*). The CV profiles of bare GCE, NiSe/GCE, VSe/GCE, and NF‐NiVSe/GCE are presented in Figure 3c. The reversible redox peaks observed are attributed to the electrochemical shuttling between [Fe(CN)_6_]^3‐^ and [Fe(CN)_6_]^4‐^ ions in bare and modified GCEs. The anodic (*I*_pa_) and cathodic (*I*_pc_) peak currents are presented in Figure 3d as a bar diagram. Notably, NF‐NiVSe/GCE demonstrated significantly greater redox activity compared to bare GCE and other modified GCEs. Moreover, the ∆*E*_p_ for NF‐NiVSe/GCE was substantially lower (170 mV) than for bare GCE (272 mV), NiSe/GCE (185 mV), and VSe/GCE (197 mV), indicating faster electron transport at the electrode/electrolyte interface. This enhanced performance is likely due to the increased surface area and abundance of active sites provided by the 3D‐flower‐like structure of NF‐NiVSe, promoting efficient electron transfer during the redox process. The CV findings are consistent with EIS results. To further investigate, the Randles–Sevcik equation (Equation (2))^[^ ^44^ ^]^ was applied to calculate the electrochemical active surface area (ECSA) of NF‐NiVSe/GCE.

(2) $$I_{p}=\left(2.69\times 10^{5}\right)n^{3/2}D^{1/2}v^{1/2}AC$$

where *I_pa_, n, D, v, A,C* denote the peak current, number of electron transfers (*n =* 1), the diffusion coefficient of [Fe(CN)_6_]^3‐/4‐^ redox probe (7.6 × 10^−6^ cm^2^ s^−1^), scan rate, active surface area, and concentration (mol cm^−3^), respectively. The ECSA of NF‐NiVSe/GCE was determined by analyzing the redox peak current at scan rates ranging from 10 to 100 mV s^−1^ (Figure 3e). A higher scan rate led to an increase in redox peak current, reflecting rapid electron transport across the NF‐NiVSe/GCE surface. The strong linear correlation between the redox peak current and the square root of the scan rate (*R* ^2^ for *I*_pa_ = 0.9997; *R* ^2^ for *I*_pc_ = 0.9995) confirmed a diffusion‐controlled electrode process (Figure 3f). From this relationship, the ECSA of NF‐NiVSe/GCE was calculated to be ≈0.21 cm^2^, nearly three times larger than that of bare GCE (0.072 cm^2^). These results highlight the superior surface area and electron transfer capabilities of the NF‐NiVSe/GCE, making it a promising candidate, as an electrode active material for electrochemical applications, including sensing and energy storage.

### 3.3 Optimizing the Electrocatalyst Loading Parameters

To achieve sensitive detection of NLT, optimizing the electrocatalyst loading parameters is crucial. The initial catalyst loading was evaluated by varying the concentration (2, 4, 6, 8, 10, and 12 mg mL^−1^) and assessing its impact on the current response of the irreversible reduction peak (R1) of NLT (100 µm) using CV in 0.5 m PB (pH 7, N_2_ saturated) at the scan rate of 50 mV s^−1^ (Figure S1a, Supporting Information). The R1 reduction peak current increased steadily with the NF‐NiVSe concentration from 2 to 8 mg mL^−1^, reaching a maximum at 8 mg mL^−1^. However, further increases in concentrations (10 and 12 mg mL^−1^) led to a decline in the reduction peak current. It could be due to the formation of a thick catalyst layer on the GCE surface, which hindered electron transfer at the electrode/electrolyte interface. Therefore, the optimal catalyst concentration was determined to be 8 mg mL^−1^, as it produced the highest cathodic peak current for NLT reduction, making it ideal for further voltammetric studies. Next, under the optimum catalyst concentration (8 mg mL^−1^), the effect of catalyst loading volume (4, 6, 8, and 10 µL) was examined using CV with 100 µm of NLT (Figure S1b, Supporting Information). Increasing the loading volume from 4 to 6 µL resulted in a higher *I*_pc_ value for the modified NF‐NiVSe/GCE electrode. However, at higher volumes (8 and 10 µL), the *I*_pc_ values decreased, likely due to the formation of an overly thick coating on the GCE surface, reducing sensitivity. Thus, 6 µL was chosen as the optimal loading volume for all subsequent voltammetric experiments. Finally, the influence of accumulation time (10, 20, 30, 40, and 50 s) of the analyte over NF‐NiVSe/GCE was investigated (Figure S1c, Supporting Information). The maximum reduction current was observed at an accumulation time of 30 s, beyond which saturation occurred in the *I*_pc_ response. Therefore, an accumulation time of 30 s was identified as the optimal condition to achieve high sensitivity for NLT detection.

### 3.4 Electrocatalytic Redox Behavior of NLT on the Modified Electrodes

**Figure 4:**
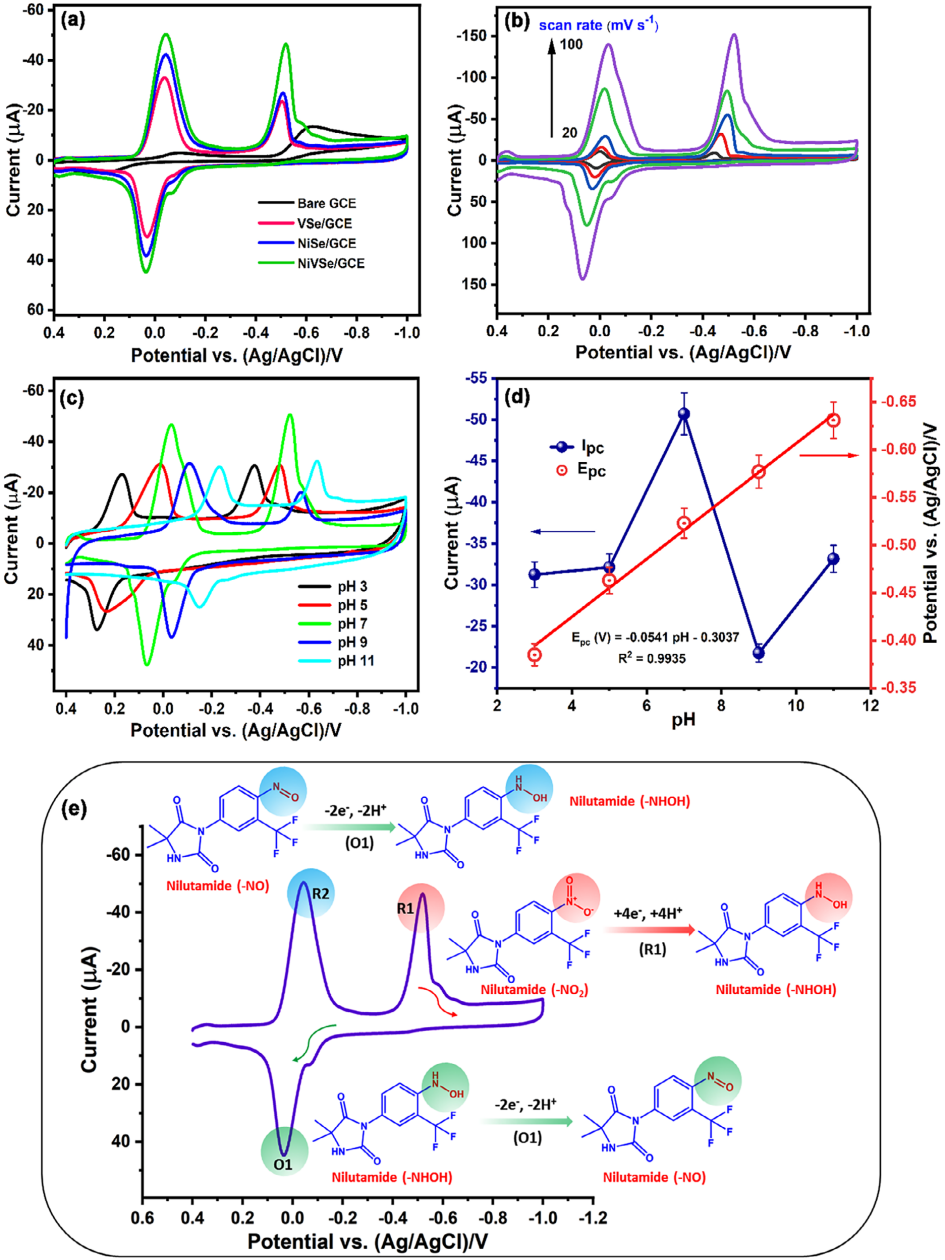
a) CV comparison of bare GCE and modified electrodes for electrochemical detection of NLT (100 µm) in PB (pH 7, N_2_ saturated) at a scan rate of 50 mV s^−1^. b) CVs of NF‐NiVSe/GCE at various scan rates (20–100 mV s^−1^) for the NLT redox reaction. c) CVs of NF‐NiVSe/GCE varying electrolyte pH (3 to 11) for NLT detection (100 µm) at 50 mV s^−1^. d) pH versus *I*_pc_ and *E*_pc_ plots based on pH variation studies. e) Proposed electrochemical redox mechanism of NLT on the NF‐NiVSe/GCE surface.

The electrocatalytic redox behavior of NLT on bare and modified electrodes was investigated using CV. NLT contains a nitro functional group (─NO_2_), which is critical for its reduction to a hydroxylamine group (─NHOH) and requires a high reduction potential. Therefore, the electrocatalytic performance of the modified electrodes was evaluated by comparing the analytes' irreversible reduction peak (R1‐NLT). CV experiments were conducted at a scan rate of 50 mV s^−1^ in PB (pH 7, N_2_ saturated) containing 100 µm of NLT, using bare GCE, NiSe/GCE, VSe/GCE, and NF‐NiVSe/GCE, to explore the reduction process from ─NO_2_ to ─NHOH and the results are shown in **Figure** **4**a. The bare GCE displayed a weak reduction peak current (*I*_pc_ (R1‐NLT) = −14.14 µA), indicating poor electron transfer efficiency. In contrast, when the GCE was modified with NiSe and VSe, significant improvements in cathodic peak currents were observed (*I*_pc_ (R1‐NFT) = −28.38 µA for NiSe/GCE; and *I*_pc_ (R1‐NLT) = −22.9 µA for VSe/GCE), which can be attributed to the enhanced catalytic activity likely due to surface defects. The use of the bimetallic 3D‐flower‐like NF‐NiVSe further enhanced the reduction response (*I*_pc_ (R1‐NLT) = −47.09 µA), underscoring the superior electrocatalytic performance of NF‐NiVSe/GCE. The R1‐NLT peak, observed during the forward scan, corresponds to the reduction of NLT's nitro group (NO_2_‐NLT) into hydroxylamine group (NHOH‐NLT) via a +4e^−^/+4H^+^ transfer (See Figure 4e). In the reverse scan, redox peaks, O1 and R2, were observed. These correspond to the oxidation of NHOH‐NLT (−2e^−^/−2H^+^) to nitroso‐NLT (NO‐NLT) and the subsequent reduction (+2e^−^/+2H^+^) back to NHOH‐NLT, respectively.

The observed R1‐NLT peak current response of NF‐NiVSe/GCE was ≈3.3 times higher than that of bare GCE, and also exhibited a significant improvement over NiSe/GCE, and VSe/GCE. These findings highlight the superior electrocatalytic activity of NF‐NiVSe/GCE compared to the other electrodes tested. The enhanced performance of NF‐NiVSe/GCE can be attributed to several factors: i) the NF‐NiVSe nanostructures consist of layered nanopetals, arranged into a 3D architecture, ii) the 3D nanoflowers provide abundant catalytic sites, and iii) this unique structure offers high surface area and excellent electronic conductivity, facilitating rapid electron transfer between the electrode surface and the analyte molecules. Consequently, NF‐NiVSe represents an effective electrode modifier with a low detection potential, demonstrating comparable performance to other composite materials reported for the electrochemical detection of NLT.^[^ ^14^, ^45^, ^46^ ^]^

### 3.5 Influence of analyte Concentration

The electrocatalytic performance of NF‐NiVSe/GCE was evaluated with increasing concentration of NLT, ranging from 50 to 200 µm, using CV in PB (pH 7, N_2_ saturated) at a scan rate of 50 mV s^−1^. The results, presented in (Figure S2a, Supporting Information), demonstrate a progressive increase in the redox peak currents of NLT as its concentration increased. A clear linear correlation between the logarithmic values of NLT concentration and the reduction peak current (R1‐NLT) is illustrated in (Figure S2b, Supporting Information), and can be expressed by the equation: log *I*_pc_ (µA) = 0.9726 log C (µm) − 0.4554, with a correlation coefficient *R* ^2^ of 0.9852. These findings indicate that NF‐NiVSe/GCE consistently produces distinct reduction peaks even at high NLT concentrations, underscoring its remarkable electrocatalytic activity and its potential as an effective electrocatalyst for NLT detection, without experiencing electrode fouling. Furthermore, the slope value obtained from the bi‐logarithmic plot of NLT is ≈1, suggesting that the electrocatalytic process at NF‐NiVSe/GCE adheres to first‐order kinetics during NLT detection.

### 3.6 Influence of Scan Rate

The electrochemical kinetics of NF‐NiVSe/GCE were investigated by varying the scan rate from 20 to 100 mV s^−1^ in PB (pH 7, N_2_ saturated) containing 100 µm NLT (Figure 4b). The electrocatalytic performance of NF‐NiVSe/GCE was assessed by analyzing the current response of the irreversible reduction peak R1 of NLT. A consistent increase in the *I*_pc_ of NLT was observed with increasing scan rates, indicating accelerated electron transfer. The relationship between *I*_pc_ and scan rate was found to be linear, as depicted in (Figure S2c, Supporting Information), with linear regression equation of *I*_pc_ (µA) = −1.6241 *v* (mV s^−1^) + 29.223, and a high correlation coefficient (*R* ^2^ = 0.9901). The linear relationship between *I*_pc_ and scan rate produced a higher *R* ^2^ value compared to the relationship between *I*_pc_ and the square root of the scan rate, indicating a surface‐controlled process. Furthermore, the slope value derived from the log scan rate versus log *I*_pc_ plot was 0.8739, which is greater than 0.5 (Figure S2d, Supporting Information). While a slope value of 0.5 from a bi‐logarithmic plot typically indicates a diffusion‐controlled process,^[^ ^47^ ^]^ the observed slope value approaching 1 suggests that the electrochemical reduction of NLT at NF‐NiVSe/GCE is governed by an adsorption‐controlled process.

The unique 3D flower‐like architecture of NF‐NiVSe provides a high surface area, which enhances the adsorption of NLT molecules onto the electrode surface. This increased adsorption leads to improved interaction between the catalytic sites and NLT molecules, resulting in a higher redox peak current at NF‐NiVSe/GCE. To estimate the surface concentration (*Γ*) of active sites, the Brown–Anson model was applied (Equation (3)).

(3) $$I_{pc}=n^{2}F^{2}\Gamma \times Av/4RT$$

where *I*_pc_ is the cathodic peak current, *Γ* is the surface concentration, *v* is the scan rate (V s^−1^), A is the electrode surface area (0.072 cm^2^), and *n*, *F*, *R*, and *T* represent their usual physical constants. Using this model (Equation (3)), the *Γ* for NF‐NiVSe/GCE was calculated to be 3.44 × 10^−8^ mol cm^−2^, confirming a high density of active sites, which contributes to the electrode's excellent electrocatalytic performance for NLT reduction. Additionally, the Laviron theory was used to analyze the electron transfer kinetics.^[^ ^48^ ^]^ The relationship between the logarithmic of scan rate (log *v*) and cathodic peak potential (*E*_pc_) was plotted, yielding a slope of 2.303 RT/αnF for the reduction peak, as expressed by Equation (4).

(4) $$E_{pc}=E^{0}-\left\{\frac{RT}{\left(1-\alpha \right)nF}\right\}\log\left\{\frac{RTk_{0}}{\left(1-\alpha \right)nF}\right\}+\left\{\frac{RT}{\left(1-\alpha \right)nF}\right\}\log v$$

where is the number of electrons transferred, *v* is the scan rate, and *E°* is the formal potential (V) obtained by projecting the vertical axis from the *E*_p_ versus *v* curve at *v* = 0. The remaining constants (*R*, *T*, and *F*) have the usual meanings. Using this equation, the value of the electron transfer coefficient (*α*) for NLT detection was determined to be 2.06. Given that an α value of 0.5 typically indicates a fully irreversible electrode reaction, the number of electrons transferred (*n*) during the NLT reduction at the R1 peak on the surface of NF‐NiVSe/GCE is estimated to be 4.

### 3.7 Influence of pH

To gain deeper insights into the detection of NLT using NF‐NiVSe/GCE, the effect of the supporting electrolyte's pH was systematically studied via CV. The current response corresponding to the irreversible reduction peak R1 of NLT was employed as a measure of NF‐NiVSe/GCE‘s electrocatalytic performance. As depicted in (Figure 4c), CVs of 100 µm NLT were recorded in PB (N_2_ saturated) with varying pH values ranging from 3 to 11 at a scan rate of 50 mV s^−1^. Notably, an increase in the reduction peak current (R1) of NLT was observed as the pH increased from 3 to 7. However, beyond pH 7, a significant decline in current response was detected as the pH increased from 7 to 11, suggesting that higher pH levels hinder the reduction of NLT due to the pronounced cationic environment. Additionally, the reduction potential (*E*_pc_) of NLT shifted progressively toward more negative values with increasing pH, reflecting the involvement of an equivalent number of protons and electrons in the reduction process. The optimal current response for NLT was recorded at pH 7, indicating that this is the most favorable electrolyte condition for NLT detection with NF‐NiVSe/GCE. Therefore, pH 7 was chosen for all subsequent electrochemical experiments to ensure optimal detection conditions. The calibration of CV results with pH against *E*_pc_ and *I*_pc_ is illustrated in (Figure 4d). The linear regression equation for *E*_pc_ (*V*) = −0.0541 [pH] – 0.3037 with a correlation coefficient of (*R* ^2^) = 0.9935, confirms a strong linear relationship between *E*_pc_ and pH. The slope value of −53.1 mV pH^−1^ closely approximates the theoretical Nernstian value of −59 mV pH^−1^ at 25 °C, further validating the involvement of equal proton and electron transfer in the irreversible reduction of NLT (R1). A plausible electrochemical redox mechanism for NLT at NF‐NiVSe/GCE is presented in (Figure 4e).

### 3.8 Determination of NLT Using NF‐NiVSe/GCE

**Figure 5:**
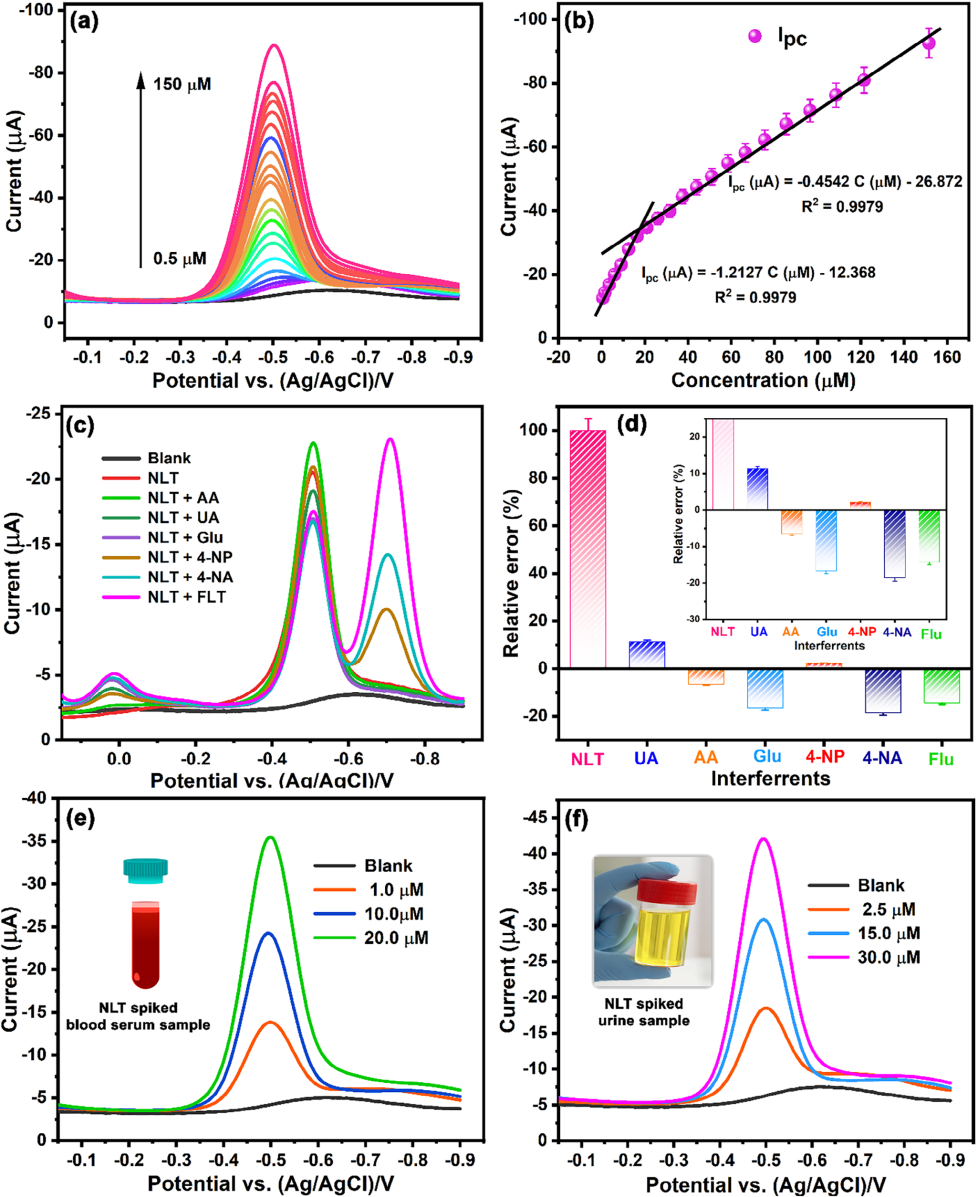
a) DPV signals of NF‐NiVSe/GCE for NLT concentrations ranging from 0.5–150 µm. b) Linear calibration plot correlating NLT concentration with reduction peak current response. c) DPV responses of NF‐NiVSe/GCE for the NLT detection in the presence of common interfering species, and d) the corresponding relative error percentage for interference analysis. DPV signals of NF‐NiVSe/GCE for real sample analysis using e) blood serum and f) urine samples, demonstrating the sensor's applicability in biological matrices.

The CV analysis demonstrates that the NF‐NiVSe/GCE significantly enhances the electrocatalytic activity during the reduction of NLT. To further assess the electrode's performance in terms of limit of detection (LOD) and sensitivity for NLT detection, the DPV technique was employed. The experiment was conducted under optimized conditions, with varying concentrations of NLT (0.5–150 µm) in PB (pH 7, N_2_ saturated) at a scan rate of 50 mV s^−1^. The electrode exhibited prompt and stable DPV signals in response to successive additions of NLT, indicating efficient electron transfer capabilities of NF‐NiVSe/GCE. The correlation between NLT concentration and peak current response is illustrated in **Figure** **5**a. Two distinct linear regression equations were obtained for different concentration ranges (Figure 5b): from 0.5 to 20 µm, the relationship is described by *I*_pc_ (µA) = −1.2127 C (µM) − 12.368 (*R* ^2^ = 0.9979), while for concentrations from 25 to 150 µm, the equation is *I*_pc_ (µA) = −0.4542 C (µM) − 26.872 (*R* ^2^ = 0.9979). Based on the slope of the calibration curve, the LOD and sensitivity were calculated using the following equations;

(5) $$\mathrm{LOD}=\frac{3\times \left(\mathrm{standard}\;\mathrm{deviation}\right)}{\mathrm{slope}\;\mathrm{value}}\;\;$$

(6) $$\mathrm{Sensitivity}=\frac{\mathrm{solpe}\;\mathrm{value}\;\mathrm{of}\;\mathrm{calbiration}\;\mathrm{plot}}{\mathrm{Surface}\;\mathrm{area}}$$

Using these equations, the LOD and sensitivity of NF‐NiVSe/GCE for NLT detection were determined to be 0.2 nm and 16.8 µA µM^−1^ cm^−2^, respectively. A comparison of these analytical parameters with previously reported NLT sensors is presented in (Table S1, Supporting Information). This comparison reveals that NF‐NiVSe/GCE offers a significantly lower LOD and higher sensitivity than existing NLT sensors. The improved electrochemical performance is attributed to the large surface area and the well‐organized 3D flower‐like structure of NF‐NiVSe/GCE.

### 3.9 Interference Study

The ability to achieve selective detection is essential to validating the functionality of a sensor. In this study, DPV was conducted under optimized conditions to evaluate the performance of NF‐NiVSe/GCE for detecting NLT in the presence of potential interfering molecules. The DPV experiment included common ions such as Ca^2+^, Na^+^, and K^+^, as well as biomolecules like glucose (Glu), uric acid (UA), ascorbic acid (AA), and sucrose (Suc) which were introduced as co‐interferents. Additionally, nitro‐containing molecules including paraoxon (PAX), Flutamide (FLU), nitrofurantoin (NFT), and 4‐nitrophenol (4‐NP) were assessed in the presence of 15 µM NLT. Figure 5c presents the DPV signals of NLT in N_2_‐saturated PB (pH 7) with a 50‐fold excess of these interfering substances on NF‐NiVSe/GCE. The results indicated that the presence of common interferents led to a minimal relative error of ≈8% in the detection of NLT (Figure 5d). However, structurally similar nitro‐group‐containing compounds exhibited reduction peaks alongside the NLT reduction peak, as shown in Figure 5d. Despite this overlap, a distinct reduction peak for NLT was consistently observed. Further evaluation of nitro‐group interferents including FLU, NFT, 4‐NP, and PAX, confirmed the selectivity of the NF‐NiVSe/GCE. The DPV results, showing that even at high interferent concentrations of interferents, the intensity of the NLT reduction peak current remained largely unchanged, and no significant shift in the reduction peak potential was detected. Although the nitro‐group interferents caused minor interference in the cathodic current response of NLT (≈15%), the sensor's selectivity for NLT remained robust. The experimental findings demonstrate that the NF‐NiVSe/GCE possesses sufficient selectivity for the detection of NLT in aqueous solution, with negligible influence from co‐interferents on the dynamic response of NLT. These results indicate that the NF‐NiVSe/GCE offers superior selectivity, making it a promising candidate for real‐time NLT sensing applications.

### 3.10 Reproducibility, Repeatability, and Stability of NF‐NiVSe/GCE

To assess the repeatability of the NF‐NiVSe/GCE for NLT (100 µm), DPV was performed using five separate electrodes. The relative standard deviation (RSD) was calculated to be ≈2.1%, indicating excellent consistency across the electrodes. Minor variations in the reduction peak current response were observed, likely attributable to handling errors (Figure S3a, Supporting Information). To evaluate the reproducibility of the method, ten consecutive measurements of 100 µm NLT were conducted using a single NF‐NiVSe/GCE electrode. The RSD of 2.5% suggested a slight decrease in the current response, potentially due to electrode saturation (Figure S3b, Supporting Information). Additionally, the stability of the modified electrode was tested by storing it at 4 °C for 30 days, followed by periodic DPV measurements of NLT under identical conditions. The current response remained stable, with an RSD of 3.7% across multiple measurements (Figure S3c, Supporting Information). This result underscores the electrode's durability over time. The structural integrity of NF‐NiVSe was examined using FE‐SEM analysis before and after extensive electrochemical testing. For the electrochemical stability test, the NF‐NiVSe/GCE was subjected to 100 continuous CV scans at 50 mV s^−1^ in the presence of 100 µm NLT in PB (pH 7.0, N_2_ saturated). Prior to testing, the electrode exhibited a well‐defined 3D‐nanoflower morphology (Figure S4a,b, Supporting Information). After electrochemical scanning, a portion of the nanoflowers transformed into densely coated spheres, likely due to the strong adsorption of NLT molecules onto the surface, facilitated by the large surface area and porous structure. Despite this partial transformation, the majority of the nanoflowers retained their original morphology, confirming the structural stability of the NF‐NiVSe material. Overall, the repeatability, reproducibility, and stability results are highly promising, suggesting that the NF‐NiVSe/GCE electrode is a reliable candidate for analytical applications in NLT detection.

### 3.11 Real‐Sample Analysis

To evaluate the sensor's efficacy and reliability for detecting NLT in real biological matrices, human blood serum (Figure 5e) and urine (Figure 5f) were employed as test samples. After centrifuging the samples at 6000 rpm to remove suspended particles, the supernatants were collected, diluted with 0.5 m phosphate buffer (PB), and analyzed via DPV. Notably, the presence of serum and urine did not significantly affect the cathodic current response, indicating minimal interference from the biological matrix. To further evaluate the sensor's performance, the samples were spiked with known NLT concentrations, and their DPV responses were recorded using NF‐NiVSe/GCE.

The spiked NLT samples showed excellent recovery rates, ranging from 98% to 99.5% (Table S2, Supporting Information), confirming the sensor's accuracy in detecting NLT even in complex biological environments. In comparison, HPLC analysis was conducted on the same sample, yielding NLT recoveries between 98.5% to 99.9%. The recovery performance of the developed electrochemical sensor was comparable to that of HPLC. However, their HPLC method was time‐intensive, taking 45 min, compared to just 6 min for the electrochemical approach. Additionally, HPLC required a complex pre‐treatment process. In contrast, the newly designed electrochemical sensor based on NF‐NiVSe/GCE was simpler, more efficient, and accurate for NLT detection, without the need for extensive solvent use or lengthy sample preparation. These results demonstrate that NF‐NiVSe/GCE can reliably detect trace NLT levels in real‐time samples like blood serum and urine, with minimal interference. The high recovery rates, low cost, and simplicity of the method support its potential for clinical and diagnostic applications.

### 3.12 Electrochemical Energy Storage

**Figure 6:**
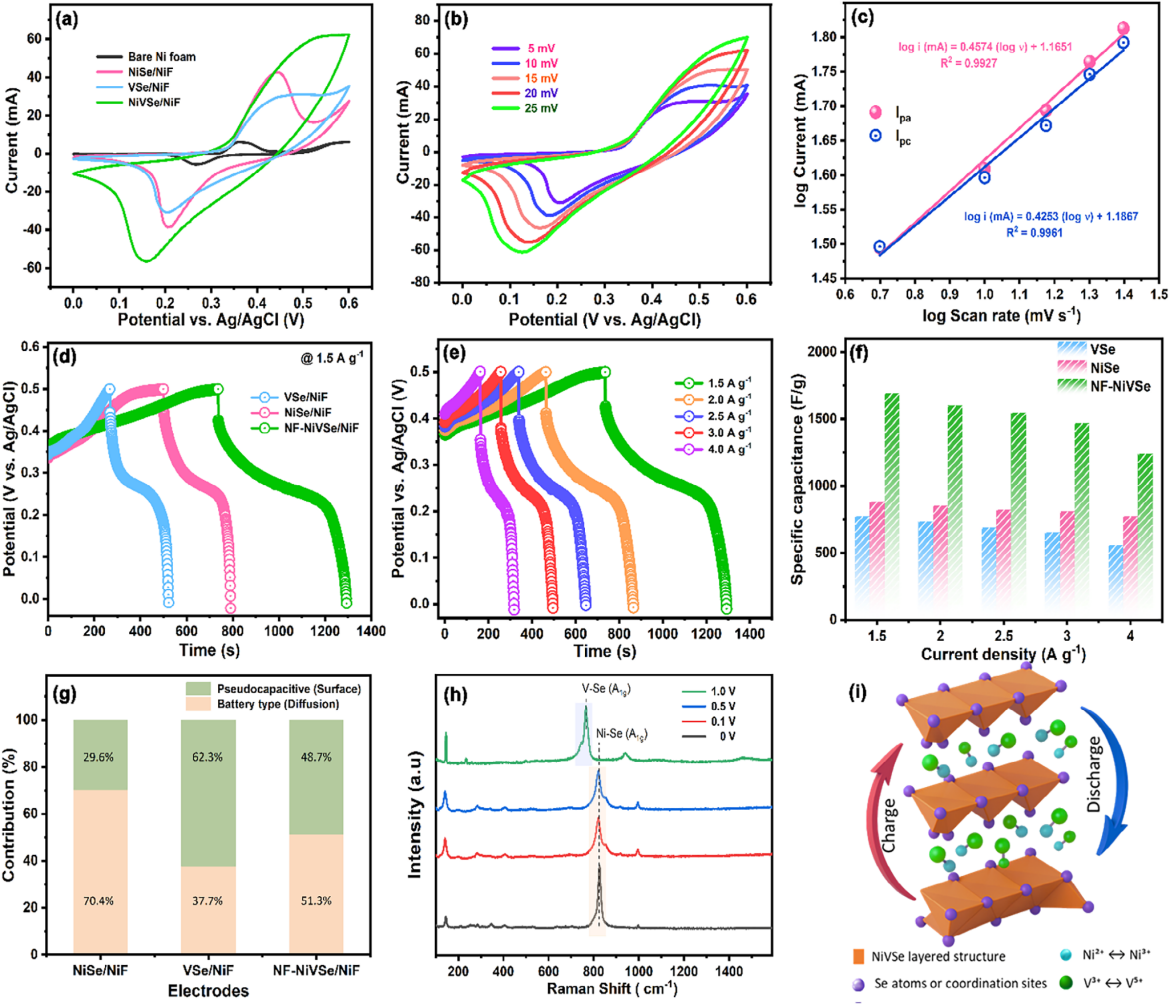
Comprehensive electrochemical and mechanistic characterization of NiSe/NiF, VSe/NiF, and NF‐NiVSe/NiF electrodes. a) CV curves at 5 mV s⁻¹ comparing the electrochemical response of bare Ni foam, NiSe/NiF, VSe/NiF, and NF‐NiVSe/NiF. b) CV curves of NF‐NiVSe/NiF at varying scan rates (5–25 mV s⁻¹), demonstrating redox behavior and scan rate dependence. c) Log–log plot of peak current versus scan rate for NF‐NiVSe/NiF, revealing diffusion‐ and surface‐controlled kinetics. d,e) Galvanostatic charge–discharge (GCD) profiles of the electrodes at 1.5 A g⁻¹ and of NF‐NiVSe/NiF at different current densities (1.5–4 A g⁻¹), respectively. f) Specific capacitance comparison as a function of current density, showing superior rate performance of NF‐NiVSe/NiF. g) Contribution of pseudocapacitive (surface‐controlled) and battery‐type (diffusion‐controlled) processes calculated via the Trasatti method, highlighting the hybrid charge storage nature of NF‐NiVSe/NiF. h) In situ Raman spectra of NF‐NiVSe/NiF at different applied potentials, indicating reversible redox activity of Ni^2^⁺/Ni^3^⁺ and V^3^⁺/V⁴⁺ through the evolution of Ni–Se and V–Se vibrational modes. i) Schematic illustration of the charge–discharge process in the NiVSe layered structure, showing dual redox activity of Ni and V centers with ion intercalation pathways contributing to both capacitive and Faradaic storage.

The electrochemical performance of bare Ni‐foam (NiF), VSe/NiF, NiSe/NiF, and NF‐NiVSe/NiF electrodes was initially investigated using a three‐electrode configuration inga 1 m KOH aqueous electrolyte. As shown in **Figure** **6**a, the CVs of Ni‐foam, VSe/NiF, NiSe/NiF, and NF‐NiVSe/NiF electrodes were recorded at a constant scan rate of 5 mV s^−1^ within a potential window of 0 to 0.6 V. All electrodes exhibited pronounced redox peaks (anodic peak ≈0.5 V and cathodic peak near 0.2 V), indicating faradaic charge storage behavior typical of battery‐type materials.^[^ ^49^ ^]^ Among the electrodes, the NF‐NiVSe/NiF displayed a notably larger background current in its CV profile compared to VSe/NiF and NiSe/NiF. This enhancement is primarily attributed to the high surface area and unique 3D‐flower‐like structure of the NF‐NiVSe, which facilitates greater electrolyte uptake and significantly improves its electrochemical performance. Furthermore, the calculated integrated area for NF‐NiVSe (0.0351 AV) exceeded that of NiSe (0.0246 AV), VSe (0.0201 AV), and bare NiF (0.0008 AV), indicating its superior charge retention capacity. These findings position NF‐NiVSe as a highly promising active material with enhanced charge storage capabilities, resulting in higher specific capacitance.

The pseudocapacitive behavior of the NF‐NiVSe was further examined by varying the scan rates in CV studies. As shown in Figure 6b, CV profiles of the NF‐NiVSe/NiF electrode were recorded at scan rates of 5, 10, 15, 20, and 25 mV s^−1^ within a fixed potential window of 0 to 0.6 V, beyond which the oxygen evolution reaction (OER) was observed.

Therefore, the potential window of 0 to 0.6 V was selected for all scan rate analyses to avoid interference from OER. Distinct redox peaks were evident at all scan rates, highlighting the redox‐active nature of the electrode. Compared to other electrodes, such as VSe/NiF (Figure S5a, Supporting Information) and NiSe/NiF (Figure S5b, Supporting Information), the NF‐NiVSe/NiF (Figure 6b) demonstrated a significantly higher redox peak current with increasing scan rates. This suggests that NF‐NiVSe possesses lower resistance and enhanced ion diffusion during the faradic processes. The kinetic behavior of the NF‐NiVSe electrode was further analyzed using the power–law relationship from CV data (Equations S1 and S2, Supporting Information).^[^ ^50^, ^51^ ^]^

(7) $$i=mv^{n}$$

(8) logi=logm+nlogv

where *v* represents the scan rate (V s^−1^), i is the current (A), and m and n are variables. The value of *n* is obtained from the slope of the log i versus log *v* plot. Ideally, a value of *n* = 1 corresponds to a surface‐controlled process, while *n* = 0.5 indicates a diffusion‐controlled process. For NF‐NiVSe, the derived *n* values for both anodic and cathodic peaks were from 0.51 and 0.50, respectively (Figure 6c), suggesting that diffusion‐controlled processes dominate during the charge storage in this system.

### 3.13 Galvanostatic Charge Discharge and EIS Studies

Galvanostatic charge–discharge (GCD) curves for VSe/NiF, NiSe/NiF, and NF‐NiVSe/NiF electrodes were recorded at a constant current density of 1.5 A g^−1^, within the potential window of 0 to 0.5 V, and the results are compared as shown in Figure 6d. The GCD outcomes of NF‐NiVSe revealed a quasi‐symmetrical profile ascribed to the battery‐like charge storage behavior, which is consistent with the CV results. The results depict that the NF‐NiVSe has a longer charge/discharge time, implying that it can store more energy than VSe, and NiSe. It could be due to a synergistic contribution of V and Ni phases in NF‐NiVSe, and its 3D flower‐like morphology with high surface area is caused for the increase in charge/discharge time. The gravimetric capacitance of the prepared electrodes was calculated using Equation (9).

(9) $$C_{s}=I\Delta t/m\Delta v$$

where I, *Δt*, *m*, and *Δv*, refer to the current density, discharge time, loading mass of active material, and potential window for the GCD experiment, respectively. At 1.5 A g^−1^ current density, the calculated gravimetric capacitance (C_s_) of NF‐NiVSe is 1695 F g^−1^, which is 1.93, and 2.2 times higher than that of NiSe (885 F g^−1^), and VSe (777 F g^−1^), respectively. These findings once again confirm that the optimum structural characteristics can fine‐tune the electrochemical performance in charge storage. Notably, NF‐NiVSe contains a 3D‐nanoflower composed of nanopetals enables a wide contact region for quick passage of electrolyte ions across the electrode/electrolyte interface. Because of the extensive interaction of OH^−^ ions on the NF‐NiVSe surface might cause to enhance the fast‐redox reaction process.

Furthermore, the GCDs of NiSe (Figure S5c, Supporting Information), VSe (Figure S5d, Supporting Information), and NF‐NiVSe (Figure 6e) electrodes were investigated at varying current densities (1.5, 2, 2.5, 3, and 4 A g^−1^). The charge–discharge curves of all electrodes exhibited a non‐linear, quasi‐symmetric triangular shape, characteristic of pseudocapacitive behavior. At the respective current densities, the gravimetric capacitance of the NF‐NiVSe electrode was calculated as 1695, 1600, 1550, 1470, and 1240 F g^−1^, demonstrating a clear trend of decreasing capacitance with increasing current density (Figure 6f).

To elucidate the underlying charge storage mechanisms of the NiSe/NiF, VSe/NiF, and NF‐NiVSe/NiF electrodes, we employed the Trasatti method, a well‐established analytical approach that quantitatively distinguishes surface‐controlled (pseudocapacitive) and diffusion‐controlled (battery‐type) contributions. As illustrated in the comparative bar chart in Figure 6g, the NiSe/NiF electrode displays a pronounced diffusion‐controlled behavior, accounting for ≈70.4% of the total capacitance, while the remaining 29.6% arises from surface pseudocapacitance. This dominance of battery‐type characteristics is attributed to the bulk redox activity of Ni^2^⁺/Ni^3^⁺ and the sluggish ion diffusion kinetics within the NiSe matrix. While this confers relatively high energy density, the rate performance is inherently limited by the slow Faradaic kinetics. In stark contrast, the VSe/NiF electrode exhibits a significantly higher capacitive contribution of 62.3%, indicative of a surface‐dominated charge storage mechanism. This behavior is facilitated by the layered architecture of VSe, which enables facile ion access and promotes rapid surface redox reactions involving the V^3^⁺/V⁴⁺ redox couple. The high conductivity and structural openness of VSe underpin its superior rate capability, making it highly suitable for high‐power supercapacitor applications. Of particular interest is the bimetallic NF‐NiVSe/NiF electrode, which demonstrates a near‐equilibrium between capacitive (48.7%) and diffusion‐controlled (51.3%) contributions. This hybrid storage behavior stems from the synergistic interplay between Ni and V redox centers and is further amplified by the unique nanoflower‐like morphology of the electrode. The hierarchical structure maximizes the electrochemically active surface area while maintaining accessible diffusion pathways, thereby supporting both rapid surface charge accumulation and bulk Faradaic reactions. Such dual‐mode charge storage is essential for achieving high energy and power densities concurrently, a critical requirement for next‐generation hybrid energy storage systems. Overall, the Trasatti method‐based analysis underscores the impact of compositional tuning and nanoscale engineering on the electrochemical behavior of transition metal selenides. The evolution from mono‐metallic (NiSe and VSe) to bimetallic (NiVSe) systems reflects a rational design strategy that enables the tailoring of energy storage characteristics toward specific application needs. Among the studied materials, NF‐NiVSe/NiF emerges as a promising candidate that bridges the gap between high energy and high‐power performance, showcasing the potential of hybrid electrode architectures in advanced supercapacitor technologies.

To elucidate the redox dynamics underpinning the charge storage behavior of the NiVSe/NiF electrode, in situ, Raman spectroscopy was performed at various applied potentials (0, 0.1, 0.5, and 1 V vs Ag/AgCl), as illustrated in Figure 6h. The spectra reveal a systematic evolution of vibrational modes with increasing potential, offering molecular‐level insight into the electrochemical processes occurring under operando conditions. A distinct Raman band centered at ≈800–820 cm⁻¹, attributed to the Ni─Se A₁g stretching mode, intensifies progressively from 0 to 0.5 V, indicative of the reversible oxidation of Ni^2^⁺ to Ni^3^⁺. This observation is consistent with the enhanced redox activity of Ni centers during the charging process. At an applied potential of 1 V, however, the Ni─Se band is markedly attenuated or entirely suppressed. This spectral transformation is ascribed to the complete oxidation of Ni^2^⁺ to higher valence states (e.g., Ni^3^⁺ or Ni⁴⁺), which modifies the local coordination environment (Figure 6i) and reduces the Raman activity of the Ni─Se bond due to diminished polarizability. Additionally, the potential‐induced formation of Ni–O or NiOOH‐like surface phases may passivate the Ni─Se framework or induce surface reconstruction, thereby further obscuring the vibrational signature. Concurrently, a weaker shoulder observed within the ≈760–790 cm⁻¹ region is tentatively assigned to the V–Se stretching mode, suggesting the involvement of V^3^⁺/V⁴⁺ redox transitions (Figure 6i). The synchronous evolution of Ni–Se and V–Se modes across the applied potential range confirms the bimetallic redox contribution to charge storage. This dual‐site activity is in strong agreement with the capacitive–diffusion hybrid mechanism previously inferred from electrochemical analyses. A minor band near ≈1000 cm⁻¹ emerges at higher potentials and is likely associated with Se–Se vibrational coupling or second‐order phonon processes, reflecting localized lattice strain under electrochemical bias.

Importantly, the low‐frequency region (100–600 cm⁻¹) remains largely unchanged, displaying weak but stable bands associated with metal–Se bending vibrations, thereby affirming the structural robustness of the NiVSe framework. Together, these results provide compelling spectroscopic evidence of the dual Faradaic activity of Ni and V centers and underscore the electrode's reversible redox behavior and structural stability during extended cycling. This operando spectroscopic validation reinforces the efficacy of NiVSe as a high‐performance hybrid electrode capable of delivering both high energy density and long‐term cycling durability.

To further investigate the capacitive and resistive properties of the prepared electrodes, EIS measurements were conducted. The Nyquist plots for bare NiF, VSe/NiF, NiSe/NiF, and NF‐NiVSe/NiF electrodes are shown in Figure S6a (Supporting Information). In the high‐frequency region, all electrodes displayed partial semicircle, while straight lines were observed in the low‐frequency region, indicating typical capacitive behavior. The equivalent circuit model was used to fit the impedance data and analyzed using NOVA 2.1.4 software (Autolab, Metrohm). The model includes components for pseudocapacitance (*C*_p_, as CPE1), double‐layer capacitance (*C*_dl_, as CPE), charge transfer resistance (*R*_ct_), solution resistance (*R*_s_), and Warburg resistance (*W_R_*).^[^ ^52^ ^]^ The semicircle observed in the high‐frequency region reflects the *R*_ct_ values, which are influenced by the electrode's surface condition, electrical conductivity, and interaction with electrolyte ions. Among the electrodes, NF‐NiVSe exhibited the lowest *R*_ct_ value of 65.8 Ω, significantly lower than those of bare NiF (305.3 Ω), NiSe (72.4 Ω), and VSe (126.1 Ω) electrodes (Figure S6b, Supporting Information). This reduction in *R*_ct_ for NF‐NiVSe can be attributed to the optimum distribution and electronic synergy between the Ni and V phases, along with its high surface area, and 3D flower‐like morphology. These structural features enhance conductivity and facilitate charge transfer, thereby boosting the electrode's overall capacity. Additionally, the vertical straight line observed in the low‐frequency region indicates a phase angle greater than 45°, nearly parallel to the *y*‐axis, signifying efficient ion transport at the electrode surface. This characteristic further confirms the improved ion diffusion and electrochemical performance of the NF‐NiVSe electrode.^[^ ^53^ ^]^

### 3.14 Symmetric Supercapacitor Device

**Figure 7:**
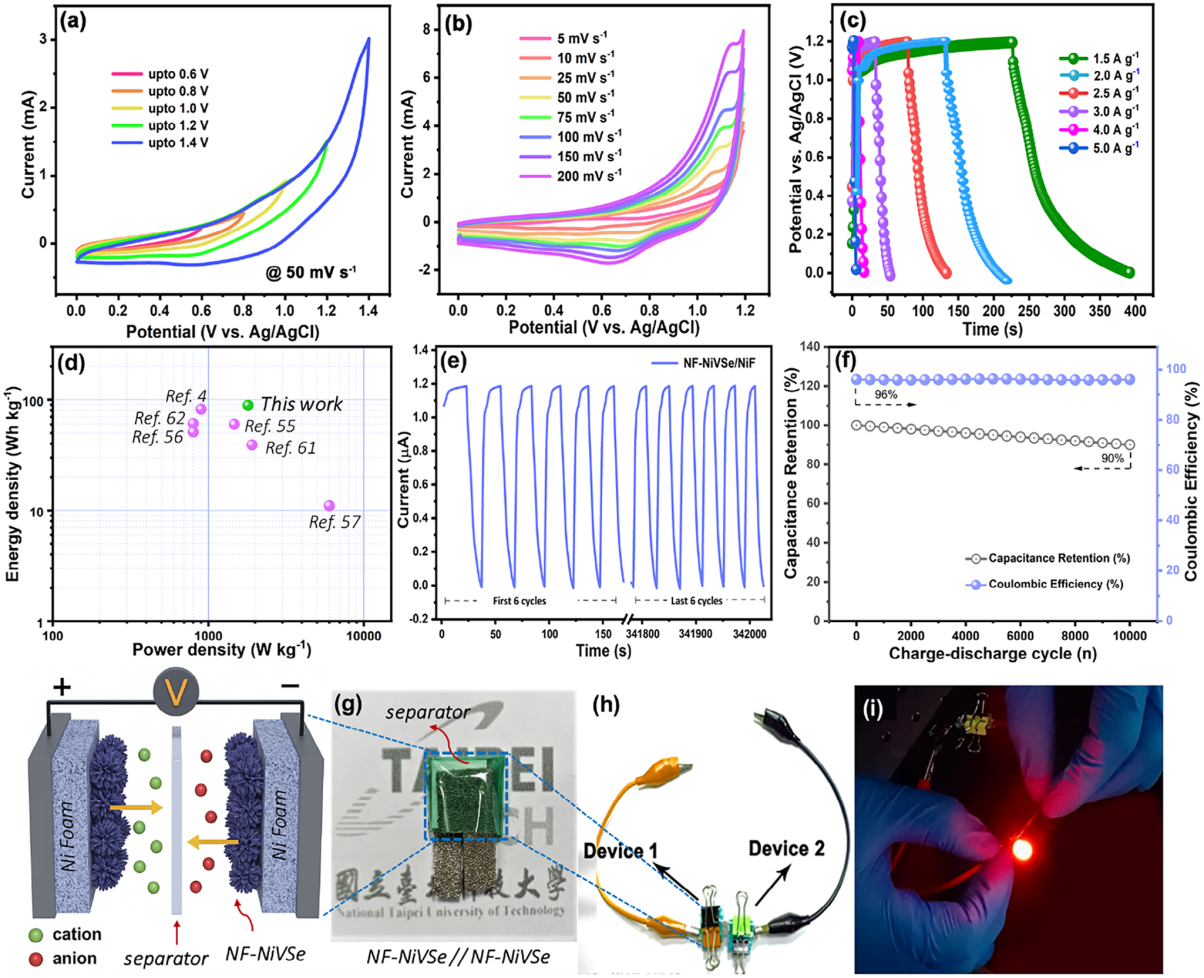
Electrochemical performance and practical application of the NF‐NiVSe‐based symmetric supercapacitor. a) Cyclic voltammetry (CV) curves at 50 mV s⁻¹ recorded for increasing potential windows, showing stable capacitive behavior up to 1.4 V. b) CV curves at varying scan rates from 5 to 200 mV s⁻¹, indicating good rate capability. c) Galvanostatic charge–discharge (GCD) profiles at different current densities (1.5–5 A g⁻¹), demonstrating high reversibility and symmetric shape. d) Ragone plot comparing the energy and power densities of the NF‐NiVSe//NF‐NiVSe device (green point) with previously reported chalcogenide‐based systems (pink points), revealing competitive energy density of 85.2 Wh kg⁻¹ at a power density of 1800 W kg⁻¹. e) Long‐term cycling performance over 10 000 GCD cycles at 5 A g⁻¹, showing consistent current profiles. f) Capacitance retention (≈90%) and coulombic efficiency (≈96%) over 10 000 cycles, confirming excellent cycling stability. g) Schematic illustration of the symmetric supercapacitor device using Ni foam current collectors decorated with NiVSe nanoflowers and separated by a polymer membrane. h) Photograph of the assembled flexible NF‐NiVSe//NF‐NiVSe device. i) Demonstration of practical applicability, where the device powers a red LED, validating its potential for real‐world energy storage applications.

To leverage the superior structural and electrochemical performance of NF‐NiVSe, an aqueous symmetric supercapacitor (SSC) device was constructed. The device utilized battery‐type NF‐NiVSe electrodes as both the anode and cathode (NF‐NiVSe//NF‐NiVSe), separated by a piece of Whatman filter paper soaked in 1 m KOH electrolyte. The electrochemical behavior and performance of the SSC device were thoroughly investigated. The working potential window was determined using CV at a fixed scan rate of 50 mV s^−1^ within voltage windows of 0 to 0.6, 0.8, 1, 1.2, and 1.4 V, as shown in **Figure** **7**a. The CV results clearly demonstrate the battery‐type redox behavior of NF‐NiVSe electrodes, with an increase in current response as the potential window expands. However, beyond 1.2 V, the curves indicate the onset of the oxygen evolution reaction (OER), leading to a deviation from stability. Therefore, the optimal operating potential window for the SSC device was set to 0–1.2 V. The interconnected flower‐like microstructure of NF‐NiVSe plays a crucial role in enhancing the electrochemical performance. This structure offers abundant active sites for electrolyte ion accumulation, thereby facilitating ion transport and extending the operational voltage window. To further evaluate the electrochemical kinetics, CV measurements were conducted at scan rates ranging from 5 to 200 mV s^−1^ within the optimized potential window of 0–1.2 V (Figure 7b). The CV curves exhibit nearly identical shapes, maintaining prominent redox peaks across all scan rates. This observation indicates excellent reversibility, fast charge transfer capability, and the ability of NF‐NiVSe electrodes to deliver stable electrochemical performance at high scan rates. GCD tests were performed at current densities of 1.5, 2, 2.5, 3, 4, and 5 A g^−1^ (Figure 7c). The GCD profiles display a nearly linear and symmetric voltage–time relationship, signifying rapid charge–discharge capability and excellent capacitive behavior. The absence of significant IR drops confirms the low internal resistance of the SSC device, further enhancing its practical applicability. The gravimetric capacitance was calculated using the GCD profiles at various current densities (Figure S7a, Supporting Information). A maximum capacitance of 423 F g^−1^ was achieved at a current density of 1.5 A g^−1^. With increasing current density, the capacitance decreased due to insufficient ion diffusion at higher rates, yet 94 F g^−1^ was retained even at 5 A g^−1^, demonstrating the excellent rate capability of the device. The energy density (*E*), and power density (*P*) of the SSC device were calculated and summarized in a Ragone plot (Figure 7d).

To assess the practical viability of supercapacitor devices, both energy density (Wh kg⁻¹) and power density (W kg⁻¹) serve as pivotal performance metrics. A comparative analysis of the data compiled in **Table** **1** highlights considerable variation in these values across diverse chalcogenide‐based electrode systems, largely governed by material composition, structural architecture, and electrolyte selection. The NF‐NiVSe//NF‐NiVSe device developed in this work delivers an impressive energy density of 85.2 Wh kg⁻¹ coupled with a high‐power density of 1800 W kg⁻¹, placing it among the most competitive systems reported to date. Notably, even at a high current density of 5 A g⁻¹, the device maintains an energy density of 11 Wh kg⁻¹ while achieving a maximum power density of 6000 W kg⁻¹, underscoring its exceptional rate capability. When benchmarked against recent high‐performance systems—such as CoNiSe₂/NC//AC (81.9 Wh kg⁻¹, 900 W kg⁻¹) and V‐doped Ni–Mo selenide/RGO//RGO (60.5 Wh kg⁻¹, 1470 W kg⁻¹)—the NF‐NiVSe device demonstrates superior energy delivery while sustaining high‐rate operability.^[^ ^4^ ^]^ Other systems, including V‐doped NiCo‐LDH//AC^[^ ^54^ ^]^ and NiCo‐LDH/(NiCo)Se₂@CC//AC,^[^ ^55^ ^]^ exhibit commendable energy densities of 60.9 and 50.6 Wh kg⁻¹, respectively, yet are limited by moderate power outputs (≈800 W kg⁻¹), reflecting a trade‐off between energy and rate performance. Conversely, Co(M)‐NiVSe//AC delivers the highest reported power density (1909.8 W kg⁻¹) among the surveyed systems, albeit with a reduced energy density of 38.7 Wh kg⁻¹, indicating an emphasis on rapid response over long‐term energy storage.^[^ ^56^ ^]^ These findings collectively underscore the balanced electrochemical performance of the NF‐NiVSe system, which effectively bridges the gap between energy‐ and power‐oriented architectures. The superior metrics are attributed to the synergistic redox activity of Ni and V centers, the enhanced ion/electron transport within the layered selenide matrix, and the structural stability of the bimetallic nanostructure. Taken together, these attributes position NF‐NiVSe as a compelling electrode material for next‐generation hybrid supercapacitors, offering an optimized combination of high energy density and fast charge–discharge capability.

**Table 1:** Comparison of electrochemical performance metrics of various chalcogenide‐based and hybrid electrode systems in different electrolytes. The NF‐NiVSe//NF‐NiVSe device reported in this work demonstrates competitive or superior performance relative to previously reported systems.

Electrode	Electrolyte	Current density [A g^−1^]	Capacitance [F g^−1^]	Energy density [Wh kg^−1^]	Power density [W kg^−1^]	Cycle/retention [%]	Refs.
Co_0.52_Ni_0.48_(OH)_2_ // AC	6 m KOH	1	179	53.4	750	10 000/97.7	[4]
Mg‐Co‐Ni LDH/rG‐9 // AC	6 m KOH	1	125	44.3	800	20 000/90.5	[5]
MnSe@CT // MnSe@CT	PVA/LiCl hydrogel	1	116.5	55.42	894.3	5000/97.1	[57]
Co_0.01_Mn_0.99_Se//Co_0.01_Mn_0.99_Se	5 m KOH	0.5	73	20.4	700.9	5000/70	[58]
PANI/Verticle Graphene/Ti electrode // PANI/Verticle Graphene/Ti electrode	0.5 m H_2_SO_4_	1	320.8	26.14	383	10 000/86	[59]
NiVSe//AC	2 m KOH	1	140.7	53.1	878.5	6000/94.4	[20]
Co(M)‐NiVSe//AC	1 m KOH	0.002	97.2	38.7	1909.8	10 000/91.6	[56]
V‐doped Ni‐Mo selenide/RGO // rGO		2	193.7	60.5	1470	10 000/82.7	[60]
V‐doped NiCo‐LDH // AC	1 m KOH	1	171.3	60.9	800	10 000/83	[54]
NiCo‐LDH/(NiCo)Se2@CC // AC	6 m KOH	1	142.3	50.6	800	8000/82.1	[55]
CoNiSe2/NC//AC	3 m KOH	1	181.7	81.9	900	6000/92.1	[61]
NF‐NiVSe//NF‐NiVSe	1 m KOH	1.5	423.4	85.2	1800	10 000/90	This work

The long‐term electrochemical performance of the NF‐NiVSe/NiF electrode was evaluated through GCD cycling over 10 000 cycles at a current density of 4 A g⁻¹, and the results are presented in Figure 7e. Figure 7e shows the GCD profiles of the first and last six cycles at a constant current, highlighting the temporal evolution of the charge–discharge curves. The electrode maintains a nearly symmetric triangular shape across all cycles, indicative of excellent reversibility and low polarization. Notably, the minimal distortion observed between the initial and final cycles suggests a high degree of structural stability and electrochemical resilience under prolonged cycling conditions.

The corresponding cycling stability and coulombic efficiency data, shown in the right panel, further affirm the robustness of the NF‐NiVSe/NiF system. The electrode retains 90% of its initial capacitance after 10 000 cycles (Figure 7f), signifying outstanding long‐term stability and minimal degradation of active sites. Simultaneously, the coulombic efficiency remains consistently high at ≈96% throughout the cycling period, indicating highly reversible charge storage with minimal parasitic reactions or leakage currents. The superior coulombic efficiency and retention performance can be attributed to the synergistic redox activity of Ni and V centers, the robust layered structure of NiVSe, and its favorable ion/electron transport properties. Furthermore, EIS analysis before and after cycling (Figure S7b, Supporting Information) revealed negligible changes in the Nyquist plot. The absence of a significant semicircle in the high‐frequency region confirms the excellent conductivity and structural integrity of the NF‐NiVSe‐based device. EDS analysis was performed to assess the compositional stability of the NF‐NiVSe electrode before and after 10 000 charge–discharge cycles (Figure S8a,b, Supporting Information). The pristine electrode exhibited a composition of Se: 64.2 wt.%, Ni: 22.4 wt.%, and V: 13.3 wt.%. After cycling, only a slight decrease in Se content to 58.7 wt.% was observed, with minor reductions in Ni and V to 20.4 and 12.5 wt.%, respectively. The appearance of 8.4 wt.% O post‐cycling is attributed to surface oxidation during prolonged exposure to alkaline electrolytes. Despite these subtle changes, the elemental distribution remains largely preserved, consistent with the electrode's excellent cycling stability (90% retention) and high coulombic efficiency (≈96%). These results confirm the chemical robustness and structural resilience of the NiVSe framework under long‐term electrochemical operation.

To demonstrate the practical viability of the SSC device, two devices were connected in series (Figure 7h). After charging the setup for 30 s, the device successfully illuminated a red LED. The LED remained illuminated for up to 6 min, as shown in the image in Figure 7i. This real‐time performance validates the superior energy storage capability of the device and its potential for use in portable electronic applications. The remarkable performance of the device implies that designing the nanocomposite NiVSe with optimum morphology could offer high surface area, porous structure, adequate structural defects, and rich ionic/electronic conductivity, and thereby it can be concluded that the NF‐NiVSe is a promising candidate for the high‐performance energy storage device.

## 4 Conclusion

In this work, we present a rationally designed bimetallic nickel vanadium selenide (NF‐NiVSe) nanostructure featuring a hierarchical 3D nanoflower morphology that integrates the structural motifs of NiSe nanoflakes and VSe nanobelts. The synergistic combination of Ni and V centers, multiple oxidation states, and interconnected conductive pathways endows NF‐NiVSe with exceptional electrochemical properties. When deployed as a sensing platform, NF‐NiVSe‐modified electrodes exhibit highly sensitive and selective electrocatalytic performance toward NLT, achieving an ultralow detection limit of 0.2 nm and near‐quantitative recovery in real biological samples. As an energy storage material, NF‐NiVSe delivers a remarkable specific capacitance of 1695 F g⁻¹ at 1.5 A g⁻¹, with 90% capacitance retention and ≈98% Coulombic efficiency after 10 000 cycles. Furthermore, the assembled symmetric NF‐NiVSe//NF‐NiVSe supercapacitor achieves an impressive energy density of 85.2 Wh kg⁻¹ at a power density of 1800 W kg⁻¹ and demonstrates practical viability by powering an LED for several minutes. In situ, Raman analysis and EIS measurements confirm the dual redox activity of Ni and V centers and reveal robust structural stability under extended cycling. These findings establish NF‐NiVSe as a multifunctional material capable of bridging high‐performance electrochemical sensing and energy storage within a unified platform. The work not only introduces a new bimetallic selenide with outstanding dual‐functionality but also provides a scalable design strategy for engineering hybrid nanostructures for advanced electrochemical applications.

## Conflict of Interest

The authors declare no conflict of interest.

## Author Contributions

R.K.D. dealt with Writing the review and editing, Writing the original draft, Methodology, Investigation, and Conceptualization. C.C.L. dealt with supervision, writing the review and editing, resources, project administration, and funding acquisition.

## Supporting information



Supporting Information

## Data Availability

Research data are not shared.
