# Supplementary material for: 3D Nanoflowers of Binary Metal‐Selenide for Improved Electrochemical Sensing and High‐Energy‐Density Energy Storage
Source: Small. 2025 Jul 11;21(37):2505860. doi: 10.1002/smll.202505860 (PMC12444850; doi:10.1002/smll.202505860)
Supplement: Supplementary file 1 — Supporting Information [file SMLL-21-2505860-s001.docx]

**Supporting Information**

**3D Nanoflowers of Binary Metal-Selenide for Improved Electrochemical Sensing and High-Energy-Density Energy Storage**

Ramadhass Keerthika Devi and Chun Che Lin*****

*Institute of Organic and Polymeric Materials, Research and Development Center for Smart Textile Technology, National Taipei University of Technology, Taipei 106, Taiwan (R.O.C).*

Corresponding author: **Chun Che Lin** ([cclin0530@mail.ntut.edu.tw](mailto:cclin0530@mail.ntut.edu.tw))

**Experimental Section**

***Chemicals and Reagents***

Ammonium metavanadate (NH_4_VO_3_); ≥99.0%, nickel chloride (NiCl_2_·6H_2_O); 99.9% trace metals basis, sodium selenate powder (Na_2_SeO_3_); 99.99% trace metals basis, nilutamide (C_12_H_10_F_3_N_3_O_4_, NLT), sodium dihydrogen phosphate (NaH_2_PO_4_), disodium hydrogen phosphate dihydrate (Na_2_HPO_4_·2H_2_O), and all the analytical grade reagents (including interfering molecules) were procured from Sigma Aldrich and used as received condition. Every precursor, analyte, nitroaromatic chemical, and biological component was used in its original form without any extra cleaning steps. A stock solution of NLT (10 mM) was prepared using ethanol. Phosphate buffer (PB) was used as an electrolyte, and its pH was adjusted as per the requirement using HCl or NaOH. All of the water-based solutions were made with deionized water (DI, 18 MΩ·cm).

***Synthesis of NF-NiVSe nanostructure***

The bi-metallic NF-NiVSe was synthesized using a hydrothermal method. To begin, a precursor solution was prepared by dissolving NH_4_VO_3_ (0.2 mmol), NiCl_2_·6H_2_O (0.2 mmol), and Na_2_SeO_3_ (0.4 mmol) in 50 mL of distilled water, followed by vigorous stirring for 10 minutes. Subsequently, 4 mL of hydrazine hydrate was added, forming a black suspension after thorough mixing. This mixture was transferred to a 100 mL Teflon-lined autoclave and subjected to hydrothermal treatment at 180 °C for 12 h. Once the reaction was complete, the autoclave was cooled to room temperature. The resulting black precipitate was washed thoroughly with ethanol and water and dried at 50 °C overnight, yielding NF-NiVSe. For comparison, monometallic NiSe and VSe were synthesized using the same procedure, with the respective nickel and vanadium precursors. These monometallic samples were evaluated for their electrochemical and structural properties to highlight the advantages of the bimetallic NF-NiVSe composition.

***Fabrication of NF-NiVSe modified electrode for electrochemical sensing of NLT***

Glassy carbon electrode (GCE; 3 mm Dia) was polished with alumina slurry to achieve a mirror sheen before being modified. Then, exactly 5 mg of DyV MFs was blended in 1 mL of isopropanol and sonicated for 30 minutes. A drop of NF-NiVSe aliquot was carefully coated over the polished GCE surface at 6 µL and dried in an air oven (50 °C). The modified GCE was denoted as NF-NiVSe/GCE, which was then used in electrochemical experiments. The detection of NLT was carried out at potentials spanning from 0.4 to -1.0 V using cyclic voltammetry (CV) and differential pulse voltammetry (DPV) techniques. All solutions were deoxygenated with a high-purity (99.99%) nitrogen (N_2_) flow before each electrochemical test was performed to reduce or eliminate the influence of free oxygen molecules in the buffer solution.

***Characterization and electrode fabrication for supercapacitor studies***

The crystallinity of the samples was studied by powder XRD (D/max-2400, Rigaku, Ultima IV) using a Cu Kα source. XPS analysis was performed via Veresprobe II spectrometer and the pattern was filed using Al Kα radiation. Surface morphology was imaged via FE-SEM (JEOL-JSM7610F), and high-resolution TEM (JEOL 2100F). The N_2_ sorption studies were performed using a 3 Flex Micromeritics-Tristar II plus. For the electrode preparation of supercapacitor studies, synthesized active materials (80 wt%), carbon black (15 wt%), and polyvinylidene fluoride (PVDF, -(C_2_H_2_F_2_)_n_-; 5 wt%) were homogenized ultrasonically in N-methyl-2-pyrrolidone (NMP, C_5_H_9_NO). The resulting black slurry was coated over Ni-foam (NF, 1 cm × 1 cm), and dried for 12 h at 80 ºC. The loaded active mass on the NF was measured to be ~1 mg/cm^2^. The electrochemical performance of bare NiF, VSe/NiF, NiSe/NiF, and NF-NiVSe/NiF electrodes (3 electrode setup), were examined using CH Instrument electrochemical workstation at ~25 ºC. Electrochemical impedance spectroscopy (EIS) was recorded between 10 mHz and 100 kHz at 0 V bias potential and 10 mV AC amplitude.

The specific capacitance (Csp; F g^-1^) of the electrodes (3 electrode system), were calculated from the discharge graph of GCD using the following equations (Eq. S1) and (Eq. S2), respectively;

$C_{sp}= \left( \frac{I \times\Delta t}{m} \right)$ (S1)

$C_{sp}= 2\left( \frac{I \times\Delta t}{m \times\Delta U} \right)$ (S2)

Where *I, ∆t,* and *m, ∆U* refers to discharge current (A), discharge time (s), loaded mass of active material, and potential window, respectively.


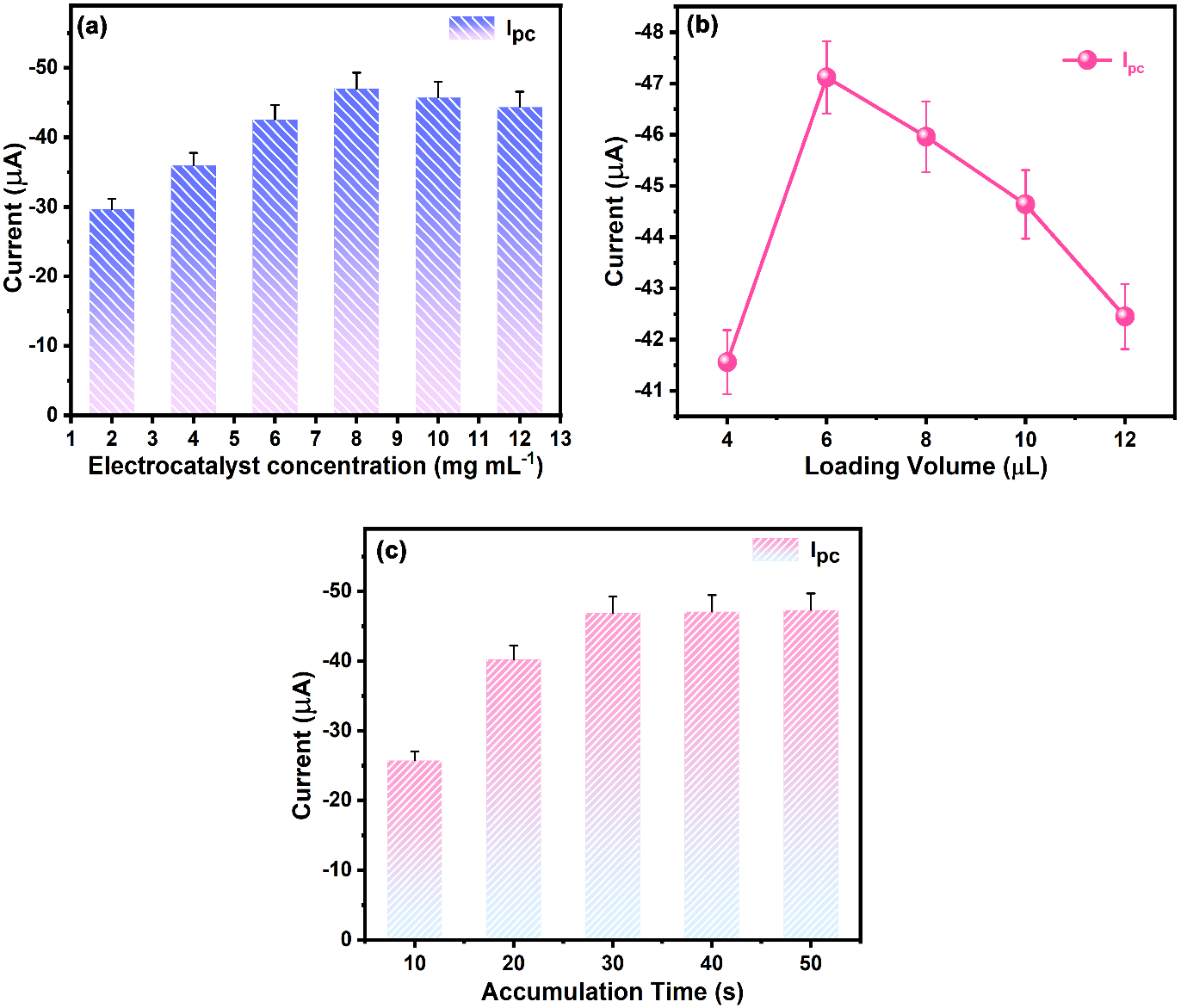


**Figure S1.** (a) Irreversible reduction peak (R1) current response of NLT (100 µM) in PB (pH 7.0, N_2_ saturated, scan rate 50 mV s^-1^) at varying loading concentrations of NF-NiVSe/GCE, demonstrating the influence of catalyst loading on the electrochemical response. (b) Current response at different loading volume of NF-NiVSe DyV MFs/GCE, highlighting the effect of electrode modification volume on detection sensitivity. (c) The impact of accumulation time of NLT on the surface of NF-NiVSe/GCE, showing insights into optimal conditions for NLT detection.

**
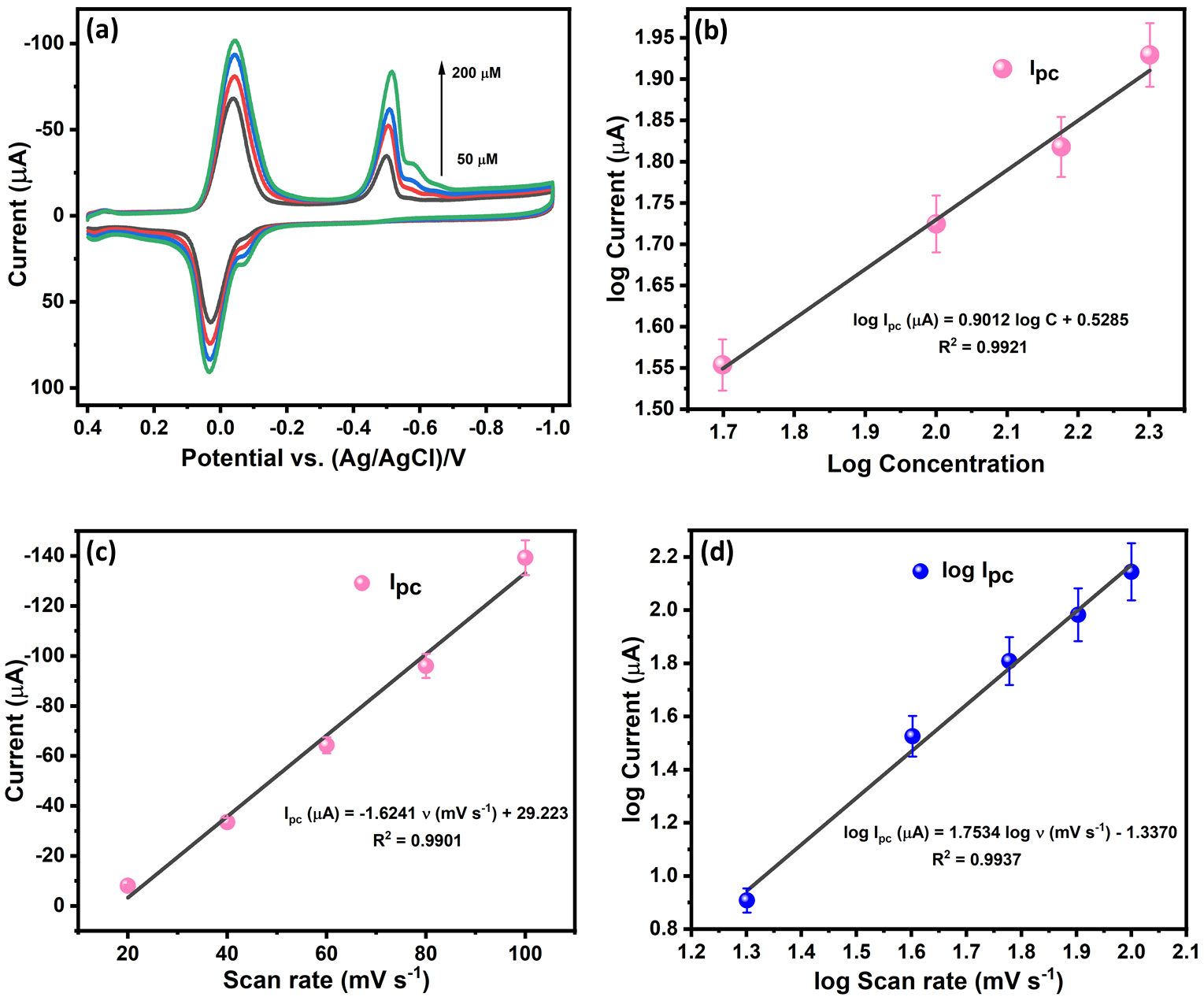
**

**Figure S2**. (a) CV responses of the NF-NiVSe/GCE at increasing concentrations of NLT (50 – 200 µM) in 0.1 M PBS (pH 7.0; scan rate 50 mV s^-1^). The CV curves exhibit a progressive increase in peak current with rising NLT concentrations, demonstrating the electrode's high sensitivity and efficient detection capability. (b) The corresponding bi-logarithmic plot of NLT concentration versus peak current, showing a clear linear trend, confirming the electrode's reliability for quantitative NLT detection. (c) Linear calibration plot between scan rate and peak current, showing a direct correlation. (d) Log-log linear calibration plot between the scan rate and peak current, further supporting the electrode’s electrochemical performance consistency across varying scan rates.


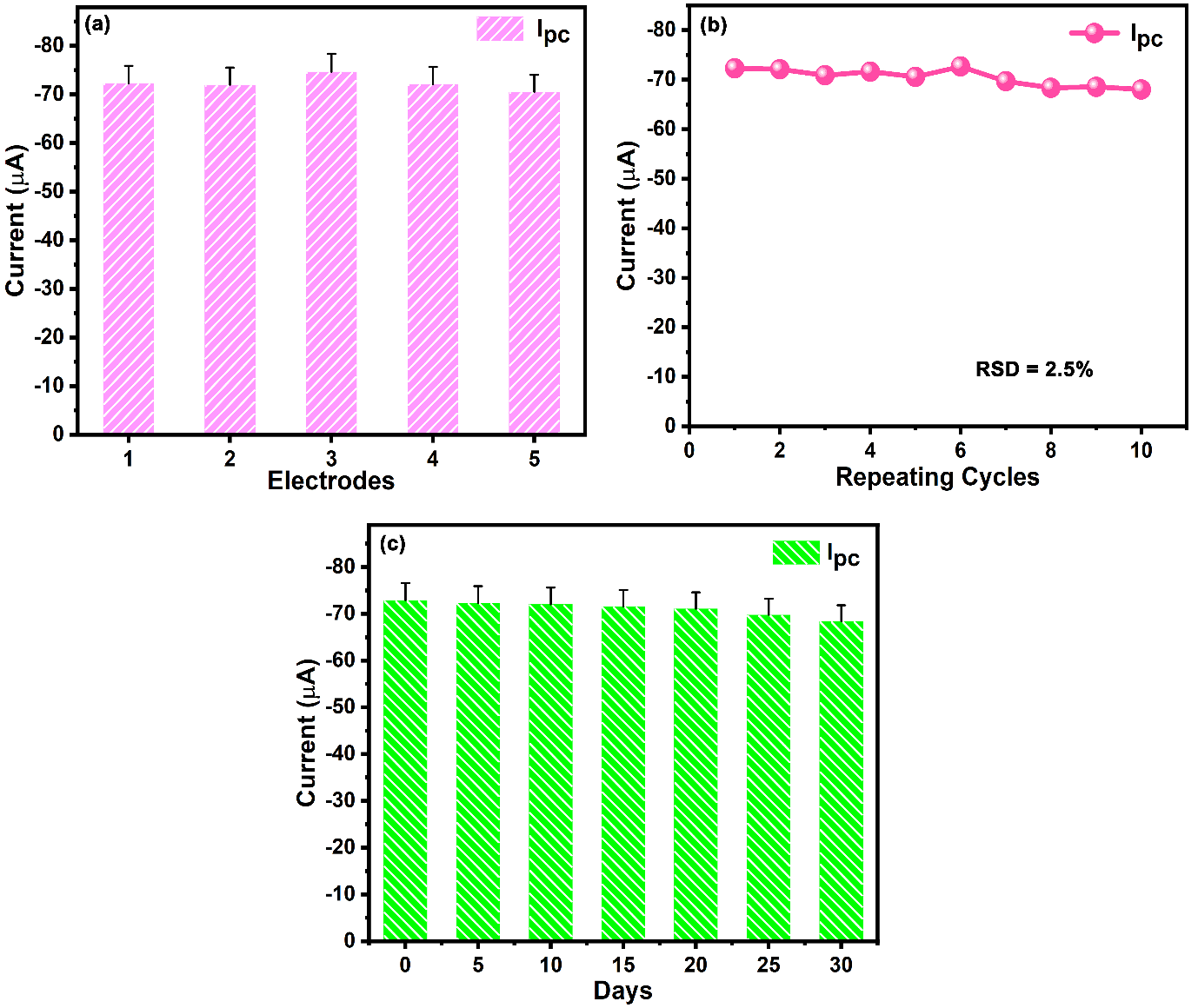


**Figure S3.** (a) DPV responses for the detection of NLT (100 µM) in 0.1 M PBS (pH 7.0; scan rate 50 mV s^-1^) using five different NF-NiVSe/GCE electrodes, demonstratine electrode consistency. (b) Current response of the NF-NiVSe/GCE under similar experimental conditions across ten repeated measurments showing excellent repeatability. (c) Storage stability results of the NF-NiVSe/GCE electrode for NLT detection over 30 days, confirming the long-term stability of the electrode for reliable performance.

**
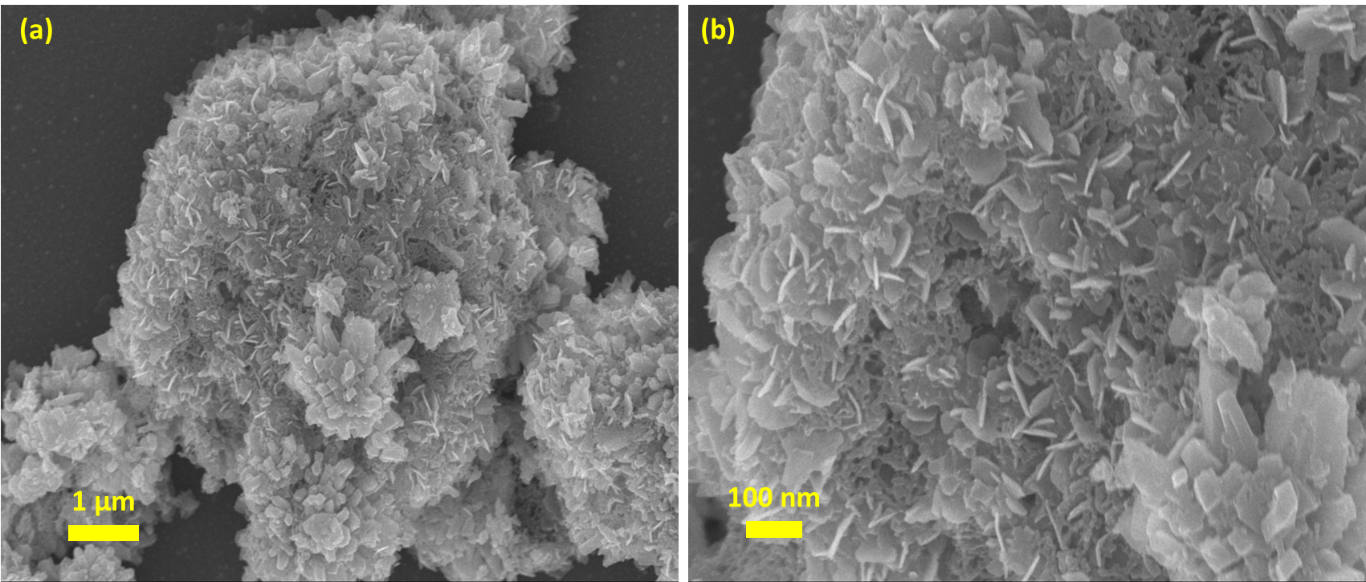
**

**Figure S4.** (a, b) FE-SEM images of NF-NiVSe/GCE after result of 100 continuous CV scans at 50 mV s^-1^ in the presence of 100 M NLT in PB (pH 7.0, N_2_ saturated). The images demonstrate the structural stability of the NF-NiVSe nanostructure, maintaining its integrity after repeated electrochemical cycling.

**
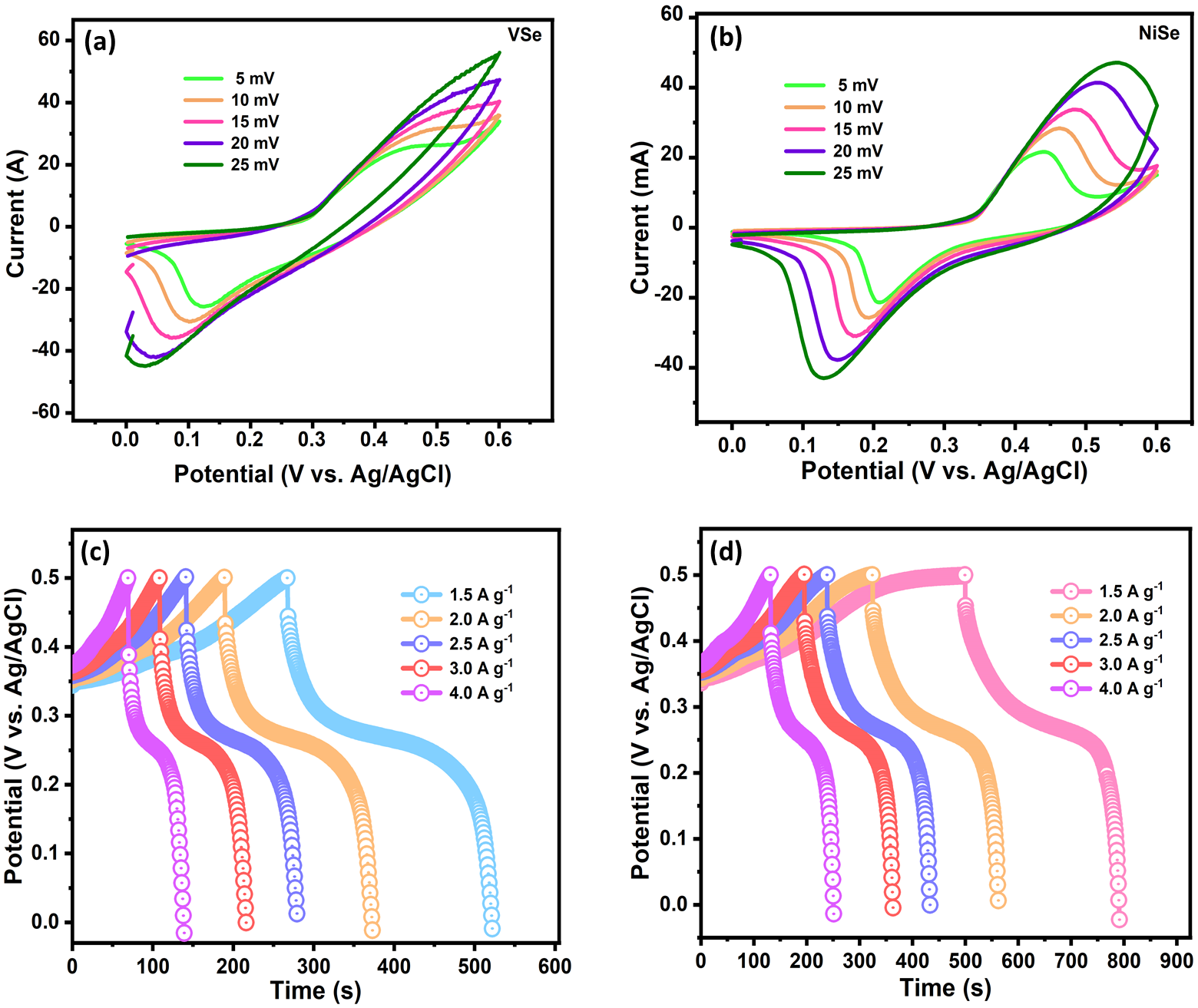
Figure S5.** CVs responses at various scan rates for (a) VSe/NiF and (b) NiSe/NiF, demonstrating the electrochemical behavior of the electrodes. Galvanostatic charge-discharge (GCD) performance at different current densities for (c) VSe/NiF and (d) NiSe/NiF, illustrating their energy storage capabilities. All supercapacitor studies were conducted in 1 M KOH solution.


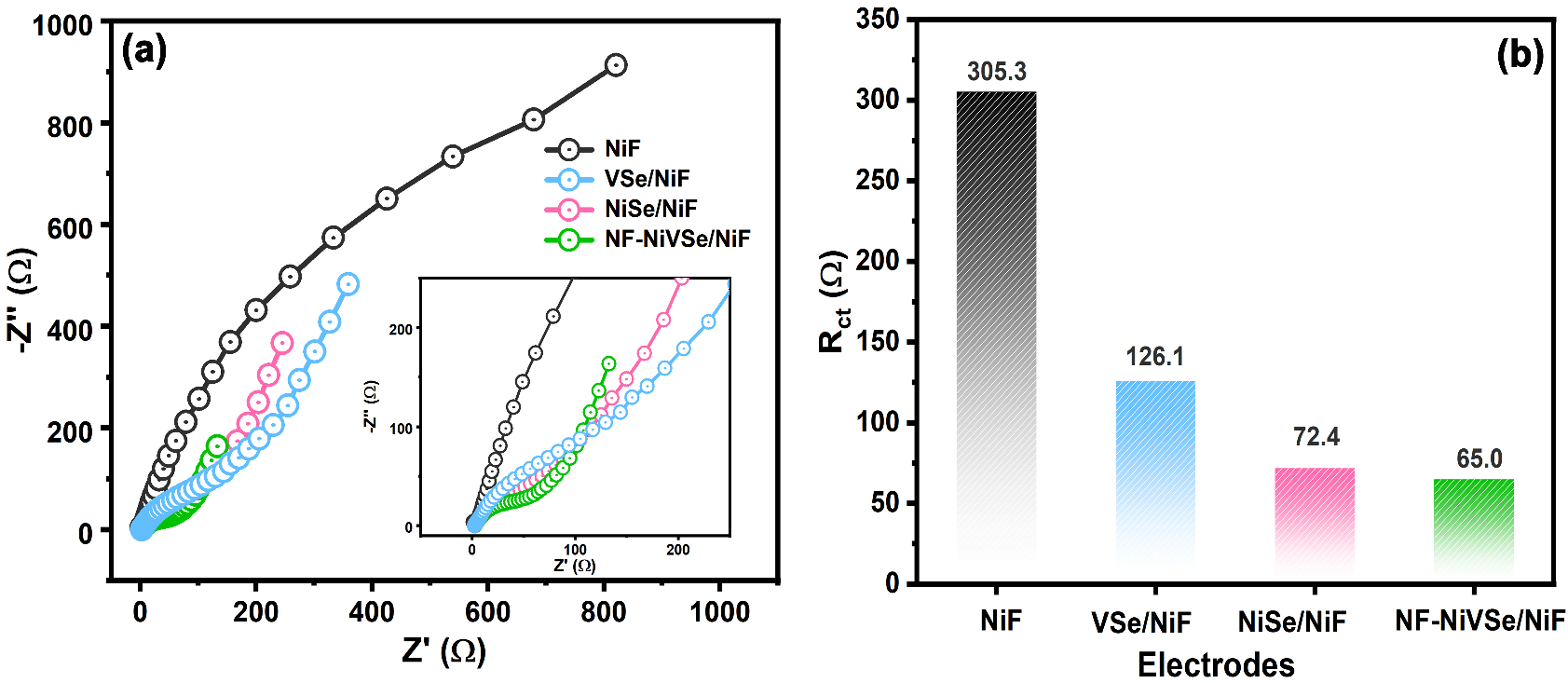
**Figure S6.** (a) Nyquist plots of NiF, VSe/NiF, NiSe/NiF, and NF-NiVSe/NiF electrodes; inset highlights the high-frequency region.(b) Comparison of charge transfer resistance (R_ct_), showing the lowest R_ct_ for NF-NiVSe/NiF (65.0 Ω), indicating superior interfacial conductivity and charge transport efficiency.

**
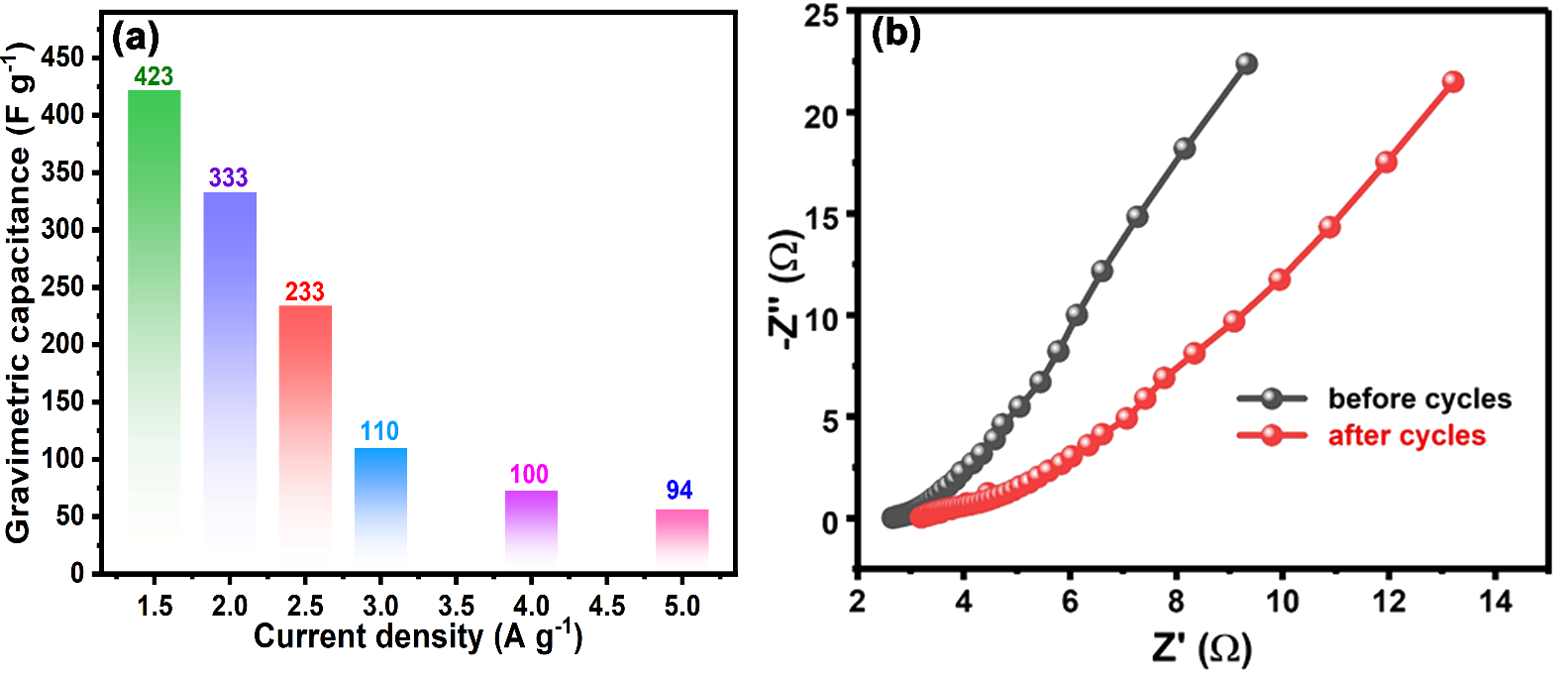
**

**Figure S7.** (a) Gravimetric capacitance versus current density plot, showing a maximum capacitance of 423 F g⁻¹ at a current density of 1.5 A g⁻¹. (b) EIS analysis before and after cycling, with minimal increase in charge transfer resistance, confirming excellent conductivity and structural stability.

**Figure S8.**
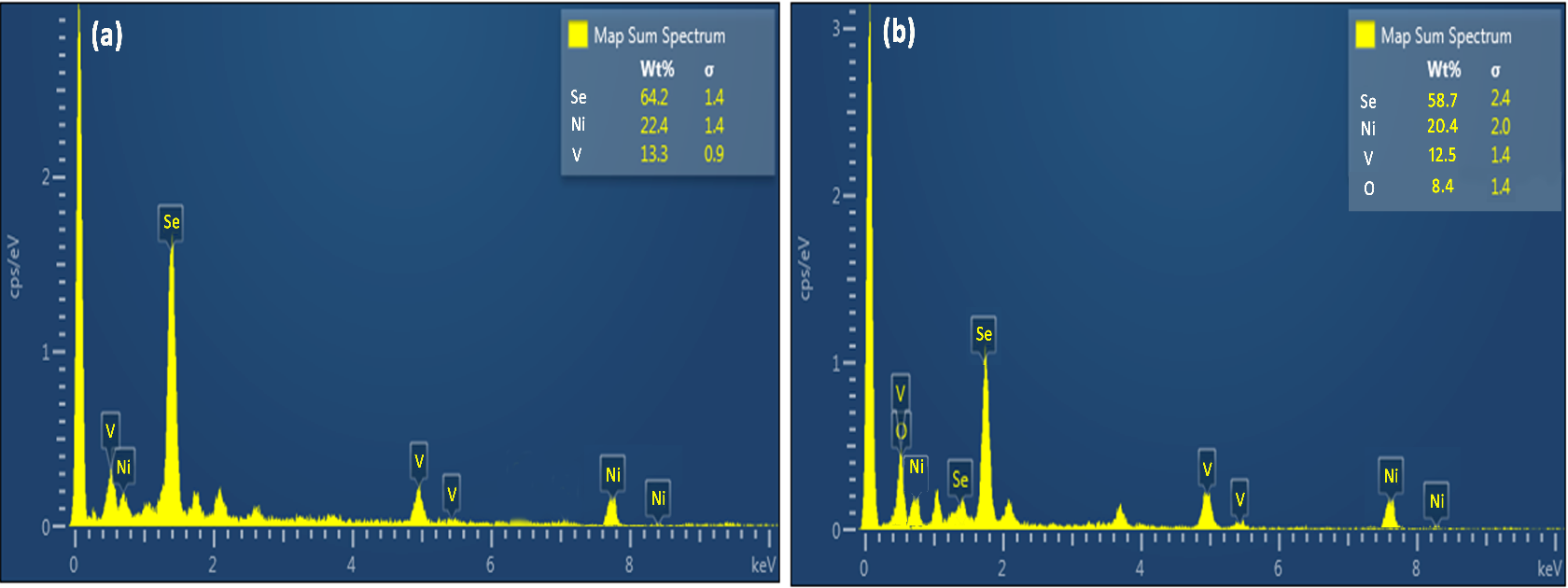
EDS spectra of NF-NiVSe electrode (a) before and (b) after 10,000 cycles. Minor reductions in Se, Ni, and V contents are observed post-cycling, with the emergence of oxygen attributed to surface oxidation. The overall compositional stability confirms the electrode's structural robustness during long-term operation.

**Table S1.** Comparison of the analytical performance of NF-NiVSe/GCE electrode for **NLT** determination with previously reported sensors.

| Materials | Linear range  (µM) | LOD  (nM) | sensitivity  (µA µM^-^¹ cm^-^²) | Ref. |
| --- | --- | --- | --- | --- |
| p-TC/hGO *^a^* | 0.05 - 158 | 1.9 | 19.4 | [1] |
| BZO/SCN *^b^* | 0.09–189.61 | 6.0 | 12.2 | [2] |
| GNR/Er_2_SnO_7_ *^c^* | 1.0 - 992 | 572 | 0.94 | [3] |
| Cu_2_V_2_O_7_/S-rGO *^d^* | 0.001–15 | 4.0 | 26.2 | [4] |
| S/P/g-C_3_N_4_ *^e^* | 0.019 – 1891 | 3.2 | - | [5] |
| SWNPs *^f^* | 0.05–318 | 2.6 | 0.92 | [6] |
| VS_4_/NCNF *^g^* | 0.001–760 | 0.09 | 6.7 | [7] |
| Cr-WO_3_ *^h^* | - | 3.1 | 49.1 | [8] |
| Sm_2_(WO_4_)_3_ *^i^* | 0.05–318 | 2.6 | 0.92 | [9] |
| β-CD-AuNP/GO *^j^* | 0.01–193 | 0.4 | 1.2 | [10] |
| NF-NiVSe *^k^* | **0.5 - 150** | **0.2** | **16.8** | **This work** |

***^a^*** partially-oxidized titanium carbide/holy graphene oxide, ***^b^*** barium zirconate on sulphur-doped graphitic carbon nitride, ***^c^*** erbium stannate integrated with graphene nanoribbons, ***^d^*** copper vanadate-sulfur doped reduced graphene oxide, ***^e^*** sulfur and phosphorus doped graphitic carbon nitride, ***^f^*** Samarium tungstate nanoparticles, ***^g^*** vanadium tetrasulfide loaded nitrogen doped carbon nanofibers, ***^h^*** chromium-doped tungsten oxide ***^i^*** Samarium tungstate, ***^j^*** β-cyclodextrin-gold nanoparticles with graphene oxide, ***^k^*** nano-flower-like nickel vanadium selenide.

**Table S2.** Recovery results of NLT in biological samples using the developed NF-NiVSe/GCE based electrochemical sensor.

| **Sample** | **Added NLT (μM)** | **Found (μM)** | **Recovery**  **(%)** | **HPLC**  **(μM)** |
| --- | --- | --- | --- | --- |
| NLT spiked blood serum | 0 | - | - | - |
|  | 1.0 | 0.93 | 93.0 ± 1.23 | 0.97 |
|  | 10.0 | 9.95 | 99.5 ± 1.82 | 9.92 |
|  | 20.0 | 19.93 | 99.6 ± 2.12 | 19.95 |
| NLT spiked urine | 0 | - | - | - |
|  | 2.5 | 1.91 | 99.1 ± 1.45 | 9.95 |
|  | 15.0 | 14.93 | 99.6 ± 1.89 | 19.98 |
|  | 30.0 | 29.95 | 99.8 ± 1.95 | 39.99 |

**References**

[1] R.K. Devi, M. Ganesan, T.W. Chen, S.M. Chen, A.M. Abbasi, M.A. Ali, M.S. Elshikh, J. Yu, H.Y. Chuang, B. Xu, S.K. Ravi, MXene-interdigitated Holey-graphene oxide nanocomposite for simultaneous detection of antibiotic and anticancer drugs with ultra-high sensitivity, Chem. Eng. J. 474 (2023) 145693. https://doi.org/10.1016/J.CEJ.2023.145693.

[2] B. Sriram, J.N. Baby, Y.-F. Hsu, S.-F. Wang, M. George, P. Veerakumar, K.-C. Lin, Electrochemical sensor-based barium zirconate on sulphur-doped graphitic carbon nitride for the simultaneous determination of nitrofurantoin (antibacterial agent) and nilutamide (anticancer drug), J. Electroanal. Chem. 901 (2021) 115782. https://doi.org/https://doi.org/10.1016/j.jelechem.2021.115782.

[3] A. Jayapaul, R.K. Panda, S.K. Ramaraj, L.Y. Lin, Y.F. Duann, Y.C. Lin, Y.C. Lu, W.C. Liu, C.W. Tung, R. Sakthivel, R.J. Chung, Design of a pebbles-like erbium stannate integrated with graphene nanoribbons for the anti-cancer drug nilutamide detection in water and biological samples, J. Environ. Chem. Eng. 12 (2024) 113048. https://doi.org/10.1016/J.JECE.2024.113048.

[4] T.S.K. Sharma, K.Y. Hwa, Rational design and preparation of copper vanadate anchored on sulfur doped reduced graphene oxide nanocomposite for electrochemical sensing of antiandrogen drug nilutamide using flexible electrodes, J. Hazard. Mater. 410 (2021) 124659. https://doi.org/10.1016/J.JHAZMAT.2020.124659.

[5] K.Y. Hwa, A. Santhan, A. Ganguly, T.S. Kanna Sharma, Two dimensional architectures of graphitic carbon nitride with the substitution of heteroatoms for bifunctional electrochemical detection of nilutamide, Chemosphere. 320 (2023) 138068. https://doi.org/10.1016/J.CHEMOSPHERE.2023.138068.

[6] P. Sundaresan, A. Yamuna, S.-M. Chen, Sonochemical synthesis of samarium tungstate nanoparticles for the electrochemical detection of nilutamide, Ultrason. Sonochem. 67 (2020) 105146. https://doi.org/https://doi.org/10.1016/j.ultsonch.2020.105146.

[7] K. Rajendran, T. Kokulnathan, S.-M. Chen, J.A. Allen, C. Viswanathan, H.A. Therese, Nitrogen doped carbon nanofibers loaded with hierarchical vanadium tetrasulfide for the voltammetric detection of the non-steroidal anti-prostate cancer drug nilutamide, Microchim. Acta. 186 (2019) 141. https://doi.org/10.1007/s00604-019-3251-x.

[8] M.M. Shanbhag, S.S. Kalanur, A.N. Alodhayb, N.P. Shetti, Porous Nanostructured Chromium-Doped Tungsten Oxide Electrocatalysts for Flutamide and Nilutamide Detection, ACS Appl. Nano Mater. 7 (2024) 9635–9648. https://doi.org/https://doi.org/10.1021/acsanm.4c01143.

[9] P. Sundaresan, A. Yamuna, S.M. Chen, Sonochemical synthesis of samarium tungstate nanoparticles for the electrochemical detection of nilutamide, Ultrason. Sonochem. 67 (2020) 105146. https://doi.org/10.1016/J.ULTSONCH.2020.105146.

[10] R. Karthik, N. Karikalan, S.-M. Chen, P. Gnanaprakasam, C. Karuppiah, Voltammetric determination of the anti-cancer drug nilutamide using a screen-printed carbon electrode modified with a composite prepared from β-cyclodextrin, gold nanoparticles and graphene oxide, Microchim. Acta. 184 (2017) 507–514. https://doi.org/10.1007/s00604-016-2037-7.

***************************************************************************
